# Polar Flagellar Biosynthesis and a Regulator of Flagellar Number Influence Spatial Parameters of Cell Division in *Campylobacter jejuni*

**DOI:** 10.1371/journal.ppat.1002420

**Published:** 2011-12-01

**Authors:** Murat Balaban, David R. Hendrixson

**Affiliations:** Department of Microbiology, University of Texas Southwestern Medical Center, Dallas, Texas, United States of America; Tufts University School of Medicine, United States of America

## Abstract

Spatial and numerical regulation of flagellar biosynthesis results in different flagellation patterns specific for each bacterial species. *Campylobacter jejuni* produces amphitrichous (bipolar) flagella to result in a single flagellum at both poles. These flagella confer swimming motility and a distinctive darting motility necessary for infection of humans to cause diarrheal disease and animals to promote commensalism. In addition to flagellation, symmetrical cell division is spatially regulated so that the divisome forms near the cellular midpoint. We have identified an unprecedented system for spatially regulating cell division in *C. jejuni* composed by FlhG, a regulator of flagellar number in polar flagellates, and components of amphitrichous flagella. Similar to its role in other polarly-flagellated bacteria, we found that FlhG regulates flagellar biosynthesis to limit poles of *C. jejuni* to one flagellum. Furthermore, we discovered that FlhG negatively influences the ability of FtsZ to initiate cell division. Through analysis of specific flagellar mutants, we discovered that components of the motor and switch complex of amphitrichous flagella are required with FlhG to specifically inhibit division at poles. Without FlhG or specific motor and switch complex proteins, cell division occurs more often at polar regions to form minicells. Our findings suggest a new understanding for the biological requirement of the amphitrichous flagellation pattern in bacteria that extend beyond motility, virulence, and colonization. We propose that amphitrichous bacteria such as *Campylobacter* species advantageously exploit placement of flagella at both poles to spatially regulate an FlhG-dependent mechanism to inhibit polar cell division, thereby encouraging symmetrical cell division to generate the greatest number of viable offspring. Furthermore, we found that other polarly-flagellated bacteria produce FlhG proteins that influence cell division, suggesting that FlhG and polar flagella may function together in a broad range of bacteria to spatially regulate division.

## Author Summary

*Campylobacter jejuni* is a leading cause of gastroenteritis in humans and requires amphitrichous (bipolar) flagella to promote infection of hosts. This pattern of flagellation results in a single flagellum at both poles, which is characteristic of many *Campylobacter* species, but fairly unusual amongst other motile bacteria. In this work, we discovered an unprecedented system to spatially regulate cell division that relies on the FlhG ATPase and amphitrichous flagellar biosynthesis. In addition to its role in other polar flagellates in controlling flagellar number, we discovered that FlhG influences spatial regulation of cell division in *C. jejuni*. Further analysis revealed that components of the flagellar motor and switch are required with FlhG to inhibit cell division specifically at the poles of the bacterium. These findings indicate that flagella have an additional function in *C. jejuni* beyond promoting motility, virulence, and colonization in functioning in a mechanism with FlhG to inhibit cell division specifically at poles. Furthermore, our findings suggest that the specific amphitrichous pattern of flagellar biosynthesis in this pathogen is an essential determinant for inhibiting cell division at both bacterial poles so that symmetrical cell division occurs and generates viable progenitors.

## Introduction

Due to spatial and numerical regulation of flagellar biosynthesis, bacterial species produce distinct patterns of flagellation. These regulatory mechanisms are especially evident in polarly-flagellated bacteria, which synthesize a defined number of flagella and form flagella only at bacterial poles. For many bacterial pathogens, strict spatial and numerical control of flagellar biosynthesis is essential for promoting proper motility and infection of hosts [1]–[4].

The FlhF and FlhG proteins have been implicated in spatial or numerical control of flagellar biosynthesis in polar flagellates such as *Vibrio* and *Pseudomonas* species and *Campylobacter jejuni*. Whereas *Vibrio* and *Pseudomonas* species each produce a monotrichous flagellum (a single flagellum only at one pole of a bacterial cell), *C. jejuni* and many other *Campylobacter* species produce amphitrichous (bipolar) flagella to result in a single flagellum at each pole. Although a defined mechanism has remained elusive, the FlhF GTPase appears to be required at an early step in flagellar biosynthesis to specifically influence formation of flagella at bacterial poles [4]–[7]. FlhG, a member of the ParA superfamily of ATPases, is involved in numerical regulation of monotrichous flagellar biosynthesis in *Vibrio* and *Pseudomonas* species, presumably by a mechanism that limits flagellar gene expression so that only one flagellum is produced per bacterial cell [1], [2].

Symmetrical cell division in bacteria also must be spatially regulated so that the divisome forms specifically at the cellular midpoint to result in two viable daughter cells of similar lengths (reviewed in [8], [9]). Without spatial regulation, the divisome may form anywhere in a bacterial cell and not always generate viable progenitors as products of cell division. Many commonly studied bacteria encode a Min system, which inhibits divisome formation at poles in *Escherichia coli* and *Bacillus subtilis*. Components of the Min system include MinD, another member of the ParA superfamily of ATPases, and MinC, the inhibitor of FtsZ polymerization into the Z-ring [10]–[15]. In *E. coli*, MinE is a topological specificity factor that spatially restricts MinCD complexes to primarily polar regions so that Z-ring formation is inhibited at poles [16], [17]. MinE stimulates the ATPase activity of MinD, which causes dissociation of MinCD complexes sequentially at each pole, resulting in polar oscillation of MinCD [14], [18]–[25]. As a result, the cellular midpoint remains relatively free of MinCD so that the Z-ring forms at the middle to promote symmetrical cell division. In *B. subtilis*, DivIVA functions as the topological specificity factor by first recruiting MinJ, which then recruits MinCD to the division site [15], [17], [26]–[28]. MinCD localizes to the divisome after a step when the Z-ring is no longer sensitive to MinC-mediated depolymerization, which likely prevents a second cell division event from occurring at the new pole of the newly formed daughter cells [15], [26], [29]. A second mechanism, termed nucleoid occlusion, also functions in *E. coli* and *B. subtilis* to inhibit Z-ring formation at the cellular midpoint [30]–[35]. Specific DNA-bound proteins inhibit Z-ring formation when the chromosomal DNA occupies the midregion of the cell. This inhibitory mechanism is relieved once chromosomal DNA is replicated and segregated to poles. Both Min and nucleoid occlusion systems may cooperatively function in many bacteria to influence formation of the divisome precisely at the midpoint at the appropriate time in a dividing cell.

The MipZ ATPase, a MinD ortholog and another ParA ATPase family member, spatially regulates Z-ring formation in *Caulobacter crescentus* [36]. Unlike MinD, MipZ itself directly dissociates FtsZ polymers and inhibits Z-ring formation. MipZ interacts with ParB, which is bound to DNA near the chromosomal origin of replication, and moves with the replicated chromosome as it segregates to the opposite pole before cell division. MipZ depolymerizes polar FtsZ polymers present from the last round of cell division, causing reorganization of FtsZ into the Z-ring near the midpoint. Interaction of MipZ with ParB-bound DNA spatially restricts MipZ to inhibit cell division primarily at poles.

Most *Campylobacter* species are amphitrichous organisms, a fairly unusual pattern of flagellation amongst polar flagellates. Flagellar motility of *C. jejuni* is an essential virulence and colonization factor required for infection of humans to result in diarrheal disease and many animals to promote commensalism [37]–[40]. Upon analysis of factors that regulate amphitrichous flagellar biosynthesis, we identified an unprecedented system to spatially regulate symmetrical cell division that involves FlhG, an ortholog of the MinD and MipZ ATPases, and components of amphitrichous flagella. We discovered that FlhG not only regulates flagellar number, but FlhG also influences where cell division occurs in *C. jejuni*. We found that deletion of *flhG* in *C. jejuni* resulted in a minicell phenotype, which is an indication of cell division occurring at polar regions rather than strictly at the cellular midpoint. Unexpectedly, mutants lacking components of the flagellar MS and C rings, which have established motor, switch, and secretory functions for the flagellum, also possessed a minicell phenotype. We propose that due to the lack of a Min system in *C. jejuni*, the flagellar MS ring and switch complex may serve as a topological specificity factor to modulate or localize a FlhG-dependent mechanism to inhibit cell division specifically at polar regions so that symmetrical division occurs to generate viable progenitors. Furthermore, our results demonstrate that amphitrichous flagellation in *C. jejuni* is not only essential for conferring motility required for infection of hosts, but also significantly influences symmetrical cell division to generate viable daughter cells. Our study also reveals that FlhG proteins of other polarly-flagellated bacteria influence placement of division sites in *C. jejuni*, suggesting that FlhG and polar flagellar biosynthesis may spatially influence cell division in a broad range of motile bacteria.

## Results

### FlhG is involved in numerical control of flagellar biosynthesis in *C. jejuni*

Members of the ParA ATPase superfamily are involved in process such as numerical regulation of flagellar biosynthesis and spatial regulation of cell division. Many polarly-flagellated bacteria appear to encode FlhG/FleN orthologs and a complete Min system including MinD (for a sequence alignment of FlhG and MinD proteins, see Figure S1). Although Min systems have not been analyzed in polar flagellates, FlhG/FleN numerically regulate flagellar biosynthesis in the monotrichous species, *V. cholerae* and *P. aeruginosa* [1], [2]. In contrast, *C. crescentus* produces the MipZ ATPase to spatially regulate cell division and does not appear to encode MinD or FlhG [36]. Completed genomes of all *Campylobacter* species encode the putative FlhG ATPase, but not MinD or any other Min proteins. Therefore, we analyzed *C. jejuni* 81–176 with an in-frame deletion within *flhG* (*Cjj81176_0101*) to ascertain a role for FlhG in flagellar biosynthesis and other processes such as cell division.

**Figure 1:**
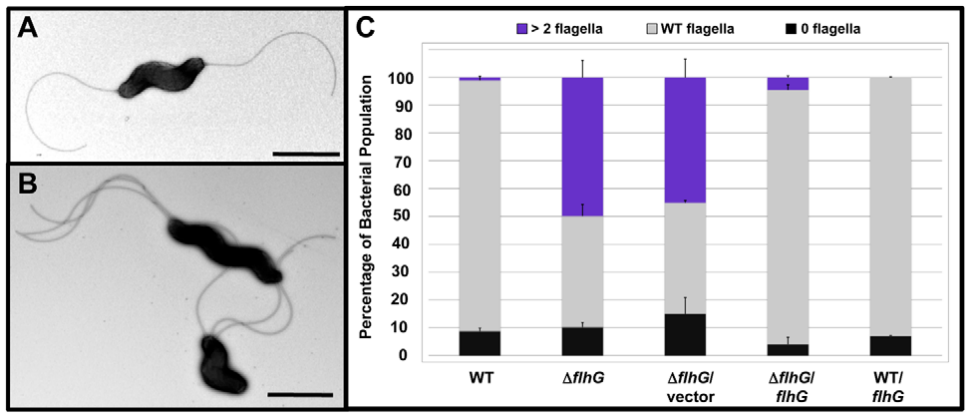
FlhG controls polar flagellar numbers in *C. jejuni*. (A and B) Electron micrographs of negatively stained (A) wild-type *C. jejuni* and (B) *C. jejuni* Δ*flhG*. Bars = 1 µm (A) and 2 µm (B). (C) Quantification of flagellar numbers of wild-type *C. jejuni* and Δ*flhG* mutant populations. Individual bacteria were analyzed for the number of flagella produced at each pole. Wild-type *C. jejuni* and *C. jejuni* Δ*flhG* were complemented with vector alone, or vector expressing wild-type *flhG*. The data are reported as the percentage of the bacterial population with the following flagellar numerical patterns: >2 flagella, producing two or more flagella at least at one pole (purple); wild-type flagella, producing a single flagellum at one or both poles (grey); and 0 flagella, aflagellated bacteria (black). Data represent the average of two experiments. Bars represent standard errors.

We first observed that FlhG numerically controls amphitrichous flagellation by examining flagellar biosynthesis of populations of wild-type *C. jejuni* and Δ*flhG* mutant strains. Over 90% of individual wild-type cells produced a single flagellum at one or both poles (62% were amphitrichous, 29% were monotrichous), which together were classified as the normal flagellar number phenotype (Figure 1A and 1C). Only about 1% of wild-type *C. jejuni* produced more than one flagellum at least at one pole. In contrast, approximately 40% of *C. jejuni* Δ*flhG* cells produced extra flagella at least at one pole, with a correlative decrease in the population producing wild-type flagellar numbers (Figure 1B and 1C). As a population, the Δ*flhG* mutant was less motile than wild-type *C. jejuni* (Figure S2A). Both motility and wild-type flagellar numbers were restored to *C. jejuni* Δ*flhG* by expressing *flhG in trans* (Figure 1C and Figure S2A).

In *V. cholerae* or *P. aeruginosa*, FlhG/FleN negatively regulates flagellar gene expression. As such, *flhG/fleN* mutants demonstrate increased expression of almost all classes of flagellar genes, which is believed to contribute to extra polar flagella [1], [2], [41]. Unlike these mutants, deletion of *flhG* in *C. jejuni* did not result in augmented expression of all classes of flagellar genes. Instead, we observed less than a 2.5-fold increase in expression of only σ^54^-dependent flagellar genes (encoding primarily rod and hook proteins), but not for other classes of flagellar genes, such as the early class encoding the flagellar type III secretion system (T3SS) or the late σ^28^-dependent *flaA* gene encoding the major flagellin (Figure S2B). These results suggest that FlhG is involved in numerical control of amphitrichous flagellation by a process different from monotrichous bacteria.

Since deletion of *flhG* resulted in an increase in the bacterial population that were hyperflagellated at least at one pole, we hypothesized that increasing the levels of FlhG in wild-type *C. jejuni* may suppress flagellation and increase the population of aflagellated bacteria. Systems to induce protein production are lacking in *C. jejuni*. Therefore, to increase FlhG levels in *C. jejuni*, *flhG* was overexpressed in wild-type *C. jejuni* by using the plasmid that complemented the *C. jejuni* Δ*flhG* mutant to restore proper flagellar numbers. However, overproduction of FlhG did not increase the aflagellated population compared to wild-type *C. jejuni* (Figure 1C).

### FlhG influences cell division

**Figure 2:**
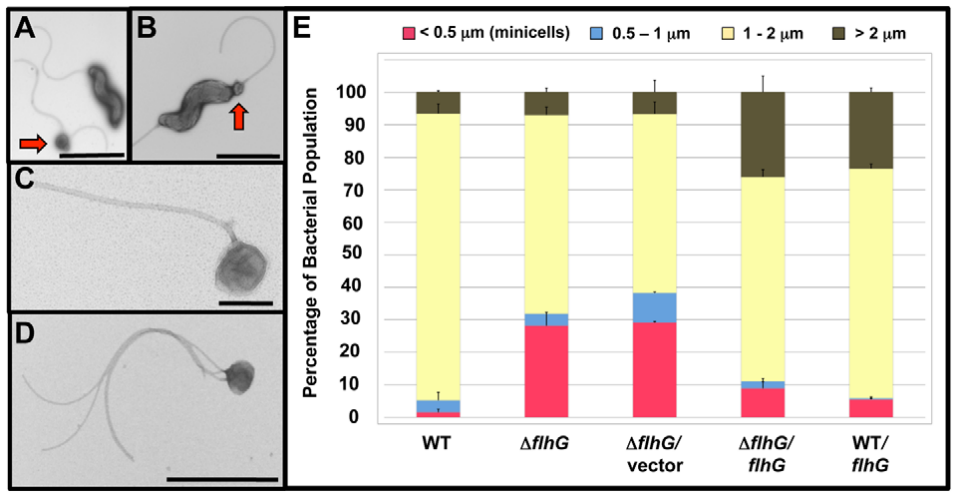
Analysis of the minicell phenotype upon deletion of *flhG* in *C. jejuni*. (A-D) Electron micrographs of negatively stained *C. jejuni* Δ*flhG* cell bodies and minicells. Bars = 2 µm (A and B), 0.2 µm (C), and 1 µm (D). Red arrows indicate minicells next to normal size bacteria (A) or forming at a pole of a bacterium (B). (E) Quantification of lengths of cell bodies of wild-type *C. jejuni* and Δ*flhG* mutant populations. The lengths of the cell bodies of bacteria were measured. Wild-type *C. jejuni* and *C. jejuni* Δ*flhG* mutant were complemented with vector alone, or vector expressing wild-type *flhG*. The data are reported as the percentage of bacterial populations with the following cell lengths:<0.5 µm, minicells (red); 0.5–1 µm (blue); 1–2 µm (yellow); and >2 µm (brown). The data represent the average of two experiments. Bars represent standard errors.

Upon closer examination of the *C. jejuni* Δ*flhG* mutant by electron microscopy, we observed a change in the distribution of lengths of the bacterial cell bodies. In addition to bacteria of normal size, minicells were abundant in the *C. jejuni* Δ*flhG* population (Figure 2A-D). The minicells were normally 0.2–0.4 µm in diameter and originated from the poles of Δ*flhG* cells (Figure 2A and 2B). Many minicells were flagellated, and some were multiply flagellated due to the increased flagellation phenotype of the Δ*flhG* mutant (Figure 2C and 2D). We were unable to determine by phase-contrast or darkfield microscopy if the flagella rotated on minicells to confer motility (data not shown). These findings indicate that minicells are most likely generated due to division occurring at poles of *C. jejuni* Δ*flhG*, similar to observations of *E. coli* and *B. subtilis minD* mutants and a *C. crescentus mipZ* mutant [16], [36], [42].

We analyzed the lengths of individual *C. jejuni* cells to assess the abundance of minicells and distribution of cell lengths within a population. Wild-type *C. jejuni* cells averaged 1.41 µm in length with approximately 88% of the population between 1–2 µm (Figure 2E). More in depth analysis revealed about 54% of the bacteria were within a narrow range of 1.1–1.5 µm (Figure S3). Only 1.6% of wild-type *C. jejuni* were minicells, which were classified as bacterial-derived, spherical particles under 0.5 µm in diameter (Figure 2E). In contrast, the minicell phenotype of *C. jejuni* Δ*flhG* was pronounced, composing 28% of the cellular population (Figure 2E). In *E. coli min* mutants, both elongated cells and minicells are present in the bacterial populations [43], [44]. However, our analysis of the *C. jejuni* Δ*flhG* mutant, did not reveal an increase in the elongated cell population. Instead, the number of bacterial cells between 1–2 µm in the Δ*flhG* population was reduced, with the average cell length of the bacterial population consequently decreased compared to wild-type *C. jejuni* to 1.12 µm (Figure 2E and Figure S3). The reason for these differences in the population composition of *E. coli* and *C. jejuni* mutants producing minicell phenotypes is currently unknown.

Complementation of *C. jejuni* Δ*flhG in trans* with wild-type *flhG* greatly reduced the minicell population to less than 9%, demonstrating that the minicell phenotype of the Δ*flhG* mutant was due to loss of *flhG* (Figure 2E). We also noticed upon addition of *flhG in trans* to either wild-type *C. jejuni* or *C. jejuni* Δ*flhG*, the elongated cell population (>2 µm) increased to 24–26%, an approximately four-fold increase relative to wild-type *C. jejuni* (Figure 2E). This elongated cell phenotype is reminiscent of *E. coli* or *B. subtilis* strains upon *minD* overexpression in the presence of the FtsZ polymerization inhibitor, MinC, or *mipZ* overexpression in *C. crescentus* [15], [16], [36]. The occurrence of minicells upon elimination of *flhG* and the elongated cell phenotype upon increased FlhG production suggest that FlhG is involved in a process to inhibit cell division in *C. jejuni*.

We next verified that minicell production in *C. jejuni* Δ*flhG* was the result of a cell division event. Cephalexin is a late-stage cell division inhibitor that targets FtsI, which is required for peptidoglycan production at a septum during the final stages of cell division [45], [46]. We monitored minicell production in wild-type *C. jejuni* and Δ*flhG* mutant strains before and after a 6-hour incubation with the highest concentration of cephalexin that caused a noticeable cell division defect without killing the bacteria. Like untreated cells, the generation time of wild-type *C. jejuni* treated with 15 µg/ml cephalexin for six hours progressed normally through 1.5–2 doublings (data not shown). However, the cephalexin-treated bacteria displayed an increase in the population of elongated cells relative to untreated bacteria (Table 1).

**Table 1:** Minicell production in *C. jejuni* Δ*flhG* upon exposure with cephalexin.

			Cell Length^a^			
Strain	Time	Treatment	<0.5 µm (minicells)	0.5–1 µm	1–2 µm	>2 µm
Wild-type	0 h	none	1.6±0.8	8.1±0.2	84.5±1.1	5.8±0.5
Wild-type	6 h	none	2.3±0.8	3.8±0.1	78.2±3.7	15.8±4.0
Wild-type	6 h	cephalexin^b^	1.5±1.5	3.9±1.4	62.9±13.6	31.8±10.7
Δ*flhG*	0 h	none	25.6±2.0	18.6±4.1	52.5±2.5	3.3±0.4
Δ*flhG*	6 h	none	31.3±2.0	6.3±0.3	57.2±4.5	5.2±2.2
Δ*flhG*	6 h	cephalexin^c^	13.8±1.1	4.0±0.9	44.8±10.1	37.4±9.9

a The long axes of the cell bodies of individual bacteria were measured. The data are reported as percentage of the populations (± standard error) that had bodies between the indicated lengths.

b The strain was grown with 15 µg/ml cephalexin for 6 hours before analysis.

c The strain was grown with 12.5 µg/ml cephalexin for 6 hours before analysis.

Upon examination of the *C. jejuni* Δ*flhG* mutant, we noticed that the mutant was more sensitive to 15 µg/ml cephalexin that wild-type cells as indicated by cell lysis that obscured confident identification of minicell production by electron microscopy. Therefore, we treated the *C. jejuni* Δ*flhG* mutant with 12.5 µg/ml cephalexin. At this concentration, the Δ*flhG* mutant progressed through two doublings similar to untreated *C. jejuni* Δ*flhG* (data not shown). Without cephalexin treatment, the minicell population only slightly increased with time compared to the Δ*flhG* culture at the beginning of the experiment (Table 1), suggesting that minicell production is fairly consistent over time. However, six hours after cephalexin treatment, the minicell population was reduced about 56% compared to untreated Δ*flhG* cells (Table 1). Furthermore, an increased number of elongated cells was observed in the cephalexin-treated Δ*flhG* cells, indicating that cephalexin influenced cell division. We interpret the data as suggesting that minicells present after cephalexin treatment were likely those present at the start of the experiment and that cephalexin largely inhibited formation of new minicells. Therefore, we conclude that minicells are formed by a process that involves cell division in *C. jejuni* Δ*flhG*.

Members of the ParA family of ATPases contain a conserved aspartic acid residue that has been proposed to be required for ATP hydrolysis, but not for ATP binding [47], [48] (Figure S1). *E. coli* MinD and *C. crescentus* MipZ mutant proteins lacking this aspartic acid are thought to be locked into an ATP-bound state that caused cell elongation due to increased inhibition of cell division [36], [49]. Analysis of this type of mutation in *E. coli* MinD, revealed an increased association of the mutant protein with phospholipids even in the presence of MinE, and a peripheral distribution of the protein along the cytoplasmic membrane [49]. This distribution allowed the protein to function with MinC to inhibit cell division throughout the cell to result in elongation. In *C. crescentus*, production of the MipZ_D42A_ mutant protein resulted in a hyperactive form of the protein that was dominant over wild-type MipZ to result in cell elongation [36]. Considering these findings, we examined a role the putative ATPase domain of FlhG in influencing cell division by mutating the similarly conserved aspartic acid residue (D61) in FlhG. To perform these experiments, we replaced chromosomal wild-type *flhG* with *flhG_D61A_*, which we predicted would encode an FlhG mutant protein locked into an ATP-bound state, which may cause increased inhibition of cell division.

**Figure 3:**
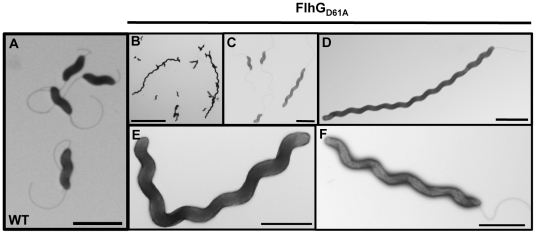
The elongated cell phenotype of *C. jejuni flhG_D61A_*. Electron micrographs of wild-type *C. jejuni* (A) or *C. jejuni flhG_D61A_* (B-F) after negative staining. Bars = 2 µm (A, C, D, and F), 10 µm (B), and 1 µm (E).

In the *C. jejuni flhG_D61A_* mutant population, we noticed a mixed population of cells, which contained cells of normal length and cells with elongated bodies (compare wild-type cells in Figure 3A with the *flhG_D61A_* mutant in Figure 3B and 3C). By analyzing the distribution of the lengths of cell bodies of the *C. jejuni flhG_D61A_* population, we found that approximately 24% of cells were elongated (>2 µm in length), compared to only 4% of wild-type *C. jejuni* (Table 2 and Figures 3D-F). Whereas elongated wild-type cells were largely confined to 2–3 µm in length, elongated *C. jejuni flhG_D61A_* cells of up to 9 µm in length were observed (Figure S4). A significant proportion of the *flhG_D61A_* population continued to produce cells of wild-type length between 1–2 µm (73.9% for *C. jejuni flhG_D61A_* vs 92.4% for wild-type *C. jejuni*; Table 2), suggesting that symmetrical cell division to produce daughter cells of normal lengths occurs with some frequency in many of these mutant cells. Of note, many *flhG_D61A_* cells produced wild-type flagella with a single flagellum at the poles and were motile as observed by darkfield microscopy (Figure 3D and 3F; data not shown). These observations suggest that the cells were metabolically active and viable.

**Table 2:** Analysis of the effects of FtsZ overexpression on minicell and elongated cell phenotypes of wild-type *C. jejuni* and *C. jejuni flhG D61A*.

	Cell Length^a^			
Strain	<0.5 µm (minicells)	0.5–1 µm	1–2 µm	>2 µm
Wild-type	1.5±0.2	1.9±1.3	92.4±2.7	4.3±1.5
Wild-type/*ftsZ* b	8.1±1.3	3.7±0.9	81.9±1.4	6.3±0.8
*flhG_D61A_*	1.0±0.3	1.2±0.2	73.9±5.8	23.9±5.5
*flhG_D61A_*/*ftsZ* b	2.3±0.2	2.8±0.9	85.9±1.4	9.0±0.8

a The long axes of the cell bodies of individual bacteria were measured. The data are reported as percentage of the populations (± standard error) that had bodies between the indicated lengths.

b The strain contained a plasmid to overexpress *ftsZ in trans*.

As observed by electron microscopy, elongated bodies of the *C. jejuni flhG_D61A_* mutant often appeared to lack septa, indicating that FlhG may function in a mechanism to inhibit divisome formation (Figure 3D-F). One of the first steps in initiating cell division is formation of FtsZ into the Z-ring. Therefore, we tested if increasing the levels of FtsZ in *C. jejuni flhG_D61A_* could overcome the apparent cell division block in these cells and reduce the cell elongation phenotype of this mutant. Similar to *E. coli* [50], overexpression of *ftsZ in trans* in wild-type *C. jejuni* caused a 5-fold increase in minicell production (Table 2), indicating that FtsZ functions in cell division. Overexpression of *ftsZ* in *C. jejuni flhG_D61A_* reduced the elongated cell phenotype of this mutant from 24% to 9% (Table 2). These results suggest a regulatory link between FlhG and FtsZ, with Z-ring formation as a potential target of cell inhibition mediated by an FlhG-dependent mechanism.

### Heterologous FlhG proteins influence cell division in *C. jejuni*

**Figure 4:**
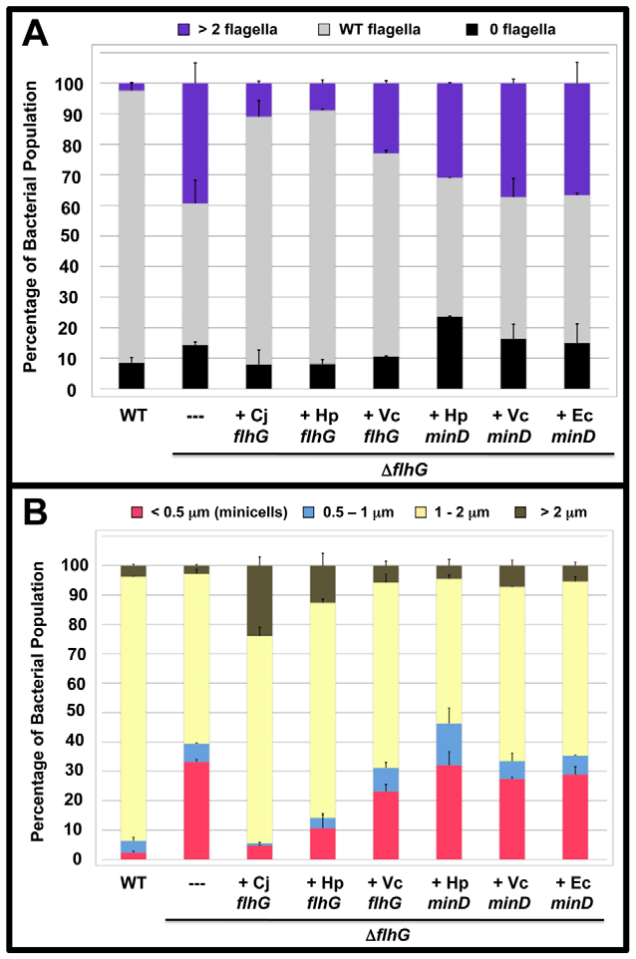
The polar flagellar number and minicell phenotypes of *C. jejuni* Δ*flhG* upon complementation *in trans* with *flhG* or *minD* orthologs. (A and B) *C. jejuni* Δ*flhG* was complemented *in trans* with vector alone (-), or *flhG* or *minD* from *C. jejuni* (Cj), *H. pylori* (Hp), *V. cholerae* (Vc), or *E. coli* (Ec). (A) Quantification of flagellar numbers of wild-type *C. jejuni* and *C. jejuni* Δ*flhG* upon complementation. Individual bacteria were analyzed for the number of flagella produced at each pole. The data are reported as the percentage of bacterial populations with the following flagellar numerical patterns: >2 flagella, producing two or more flagella at least at one pole (purple); wild-type flagella, producing a single flagellum at one or both poles (grey); and 0 flagella, aflagellated bacteria (black). The data represent the average of two experiments. Bars represent standard errors. (B) Quantification of lengths of the cell bodies of wild-type *C. jejuni* and *C. jejuni* Δ*flhG* upon complementation. The lengths of the cell bodies of bacteria were measured. Wild-type *C. jejuni* and *C. jejuni* Δ*flhG* mutant were complemented with vector alone, or vector expressing wild-type *flhG*. The data are reported as the percentage of bacterial populations with the following cell lengths:<0.5 µm, minicells (red); 0.5–1 µm (blue); 1–2 µm (yellow); and >2 µm (brown). The data represent the average of two experiments. Bars represent standard errors.

Factors that spatially regulate formation of cell division sites have not been examined in other polar flagellates. Because other polarly-flagellated bacteria produce FlhG homologs that control flagellar number, we reasoned that these FlhG proteins may have an additional capacity like *C. jejuni* FlhG to influence cell division. Therefore, we analyzed if either FlhG or MinD proteins from other polarly-flagellated bacteria or *E. coli* MinD could functionally complement *C. jejuni* Δ*flhG* for numerical control of flagellar biosynthesis or spatial control of cell division to reduce the minicell phenotype. For these experiments, heterologous *flhG* or *minD* genes were cloned into a plasmid downstream of a constitutive promoter to ensure expression. These plasmids were then used to complement *in trans C. jejuni* Δ*flhG*. We found that *H. pylori* FlhG was just as competent as *C. jejuni* FlhG in reducing extra polar flagella and restoring wild-type flagellar numbers to *C. jejuni* Δ*flhG* (Figure 4A). Furthermore, *H. pylori* FlhG dramatically reduced the minicell population of the *C. jejuni* Δ*flhG* mutant and even caused an increase in elongated cells (Figure 4B), suggesting that *H. pylori* FlhG can function in a mechanism to inhibit cell division. In addition, *V. cholerae* FlhG partially restored both wild-type flagellar numbers and normal cell division to *C. jejuni* Δ*flhG* (Figure 4A and 4B). In contrast, all MinD proteins failed to complement *C. jejuni* Δ*flhG* for either phenotype (Figure 4A and 4B). These results indicate that *H. pylori* FlhG, and to a lesser extent *V. cholerae* FlhG, have the ability to modulate cell division. Secondly, these findings suggest that *C. jejuni* has evolved to preferentially use FlhG to regulate cell division and numerically control flagellar biosynthesis.

### FlhF and the flagellar MS ring and switch complex function with FlhG to modulate cell division

**Figure 5:**
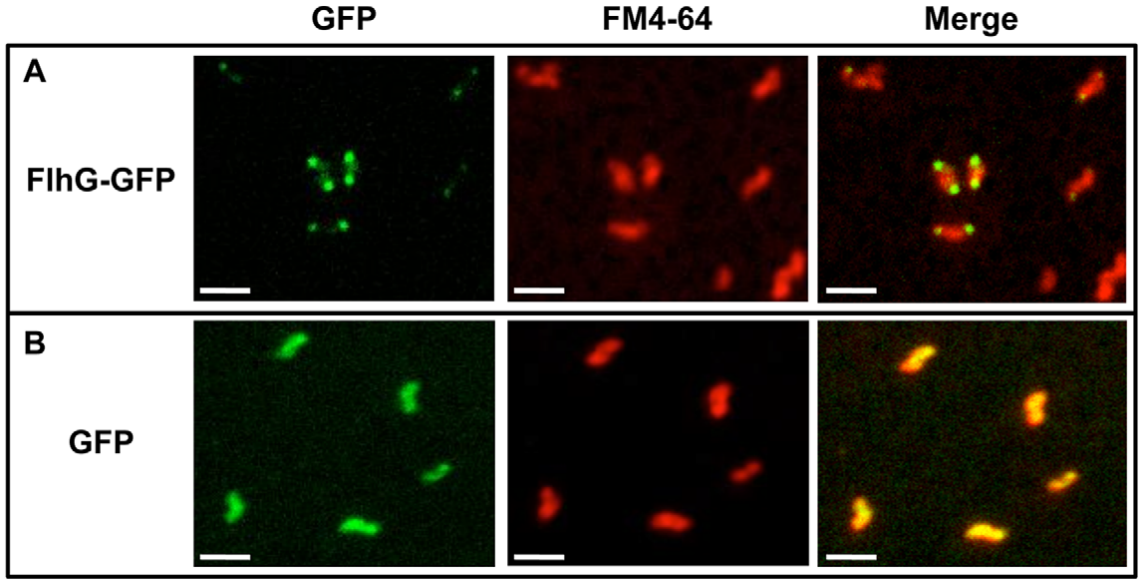
Analysis of cellular localization of FlhG-GFP. *C. jejuni* Δ*flhG* containing plasmids to express wild-type FlhG with a C-terminal GFP (A) or GFP alone (B) were stained with FM4-64 to visualize membranes. Shown are images visualizing FlhG-GFP or GFP alone (green), FM4-64 stained membranes alone (red), and merged images. Bar = 2 µm.

Our data suggest that the lack of FlhG results in the production of minicells due to polar cell division. Therefore, if FlhG is involved in a mechanism to prevent cell division at poles, it may be expected that FlhG localizes to polar regions to mediate this division inhibitory effect. We attempted to analyze the ability of FlhG to localize to poles of *C. jejuni* Δ*flhG* by using a plasmid to express *flhG-gfp*, which produces wild-type FlhG with a C-terminal GFP. The use of fluorescent protein technology to analyze cellular localization of proteins has typically been challenging in *C. jejuni* and other ε-proteobacteria. However, we were able to observe fluorescence due to FlhG-GFP in a small population of cells. In these bacteria, fluorescence was observed often at both poles, with some diffuse cellular fluorescence also visible (Figure 5). In contrast, *C. jejuni* Δ*flhG* producing GFP alone demonstrated diffuse fluorescence throughout the cell (Figure 5). These results suggest that FlhG is likely polarly localized and possibly available at poles to function in a mechanism to prevent cell division.

Because FlhG-GFP was detected primarily at poles in bacteria producing FlhG-GFP, we considered if other factors present at poles of *C. jejuni* may function with FlhG to limit cell division at poles. A leading candidate for a polarly-localized protein that may interact with FlhG is the FlhF GTPase. Previous studies suggested that the FlhF and FlhG proteins of *C. jejuni* and *Vibrio alginolyticus* interact, which may influence their respective ability to spatially and numerically control flagellar biosynthesis [4], [51]. FlhF of *C. jejuni* and other polar flagellates has been implicated as a regulatory factor required for expression of flagellar genes and to properly localize flagellar biosynthesis to poles [1], [4], [5], [7], [52]. Of note, FlhF polar localization has been observed in *C. jejuni* [53]. A current hypothesis for a role of *C. jejuni* FlhF in polar flagellar placement suggest that the GTPase activity of FlhF may influence its positioning to the new pole after cell division. After polar localization, FlhF may promote organization of the initial flagellar proteins, such as the motor, switch, and secretory components at the pole [6].

**Figure 6:**
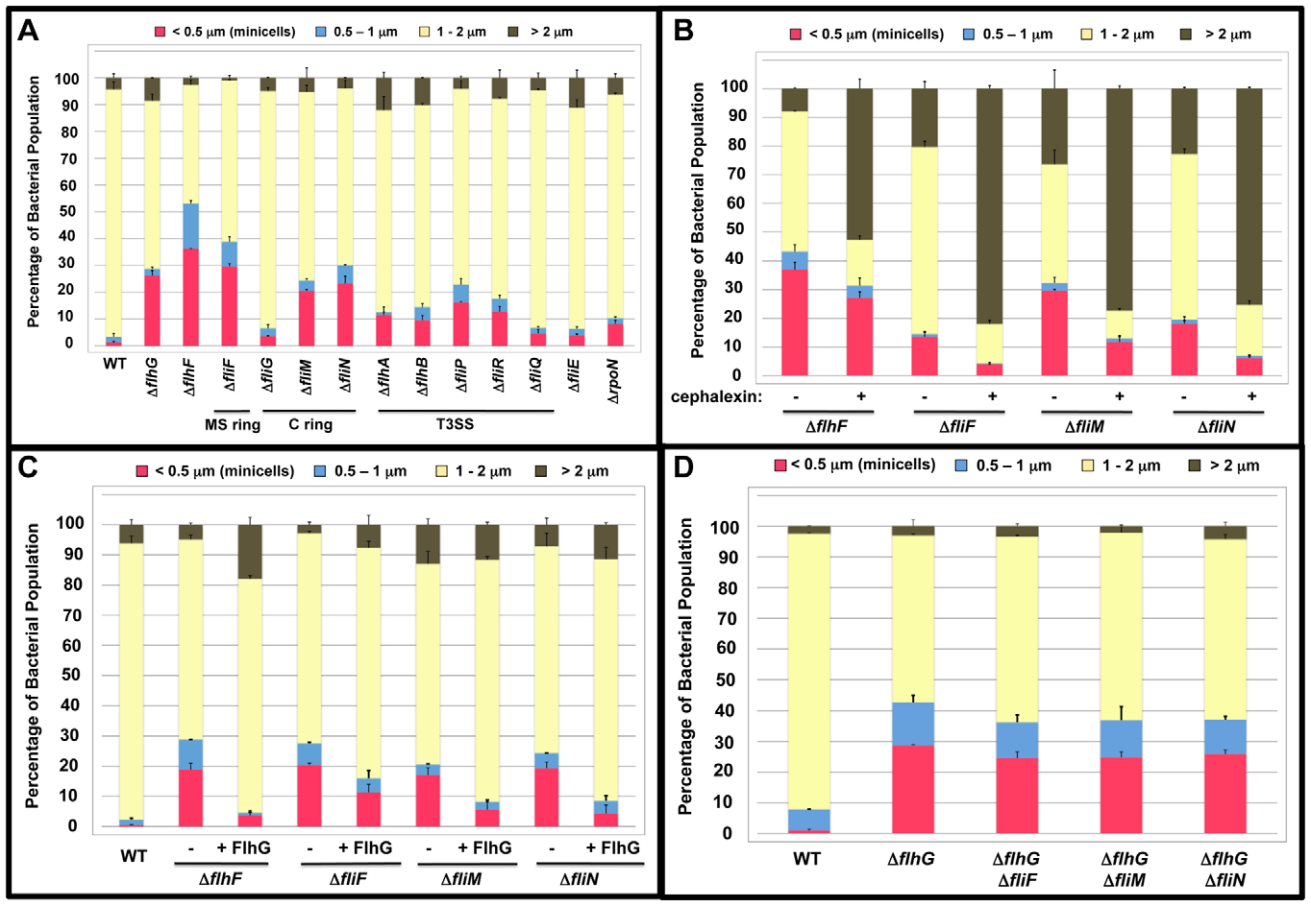
Analysis of the minicell phenotype of *C. jejuni* flagellar mutants. (A) Quantification of the lengths of the cell bodies of wild-type *C. jejuni* and mutants lacking a motility gene. (B) Quantification of the lengths of the cell bodies of mutants lacking FlhF, FliF, FliM or FliN after treatment with cephalexin. Strains were grown in liquid broth in the absence (-) or presence (+) of 15 µg/ml cephalexin for 6 h. The lengths of the cell bodies were then measured. (C) Quantification of the lengths of the cell bodies of wild-type *C. jejuni* or mutants lacking FlhF, FliF, FliM, and FliN upon overexpression of *flhG in trans*. The mutants were complemented with either vector alone (-) or a plasmid to overexpress wild-type *flhG* (+ FlhG). (D). Quantification of the lengths of the cell bodies of wild-type *C. jejuni*, *C. jejuni* Δ*flhG,* and *C. jejuni* mutants lacking *flhG* and either *fliF*, *fliM*, or *fliN*. For (A-D), the lengths of the cell bodies of individual bacteria were measured. The data are reported as the percentage of the population with the following cell lengths:<0.5 µm, minicells (red); 0.5–1 µm (blue); 1–2 µm (yellow); and >2 µm (brown). Data represents the average of two experiments. Bars represent standard errors.

Considering the potential interactions between FlhF and FlhG, we examined a *C. jejuni* Δ*flhF* mutant and observed a minicell population that was at least as prevalent as that of *C. jejuni* Δ*flhG* (Figure 6A and Figure S5). To determine if the minicell phenotype of the Δ*flhF* mutant was linked to FlhG, we expressed *flhG in trans* in the Δ*flhF* mutant to result in *flhG* overexpression due to the presence of both chromosomal- and plasmid-encoded copies of the gene. In this strain, the minicell phenotype caused by deletion of *flhF* was suppressed (Figure 6C). These results suggest that FlhF and FlhG are linked in a mechanism to influence cell division in *C. jejuni*. Furthermore, by overexpressing FlhG in the Δ*flhF* background, FlhG overcomes an apparent dependency on FlhF to modulate cell division.

Two hypotheses were developed for how FlhF may influence an FlhG-dependent mechanism to inhibit cell division at poles. First, we considered if either σ^54^-dependent flagellar gene expression or flagellar rod biosynthesis, which are both dependent on FlhF [6], [54], are required for FlhG to function in a mechanism to inhibit cell division at poles. If lack of σ^ 54^-dependent flagellar gene expression or rod biosynthesis caused the minicell phenotype in the Δ*flhF* mutant, minicell production in a Δ*rpoN* mutant (which lacks σ^ 54^) or a Δ*fliE* mutant (which lacks rod biosynthesis) would be expected. However, neither mutant demonstrated a significant minicell phenotype compared to the Δ*flhG* or Δ*flhF* mutants (Figure 6A).

For the second hypothesis, we considered if flagellar components, which are likely dependent on FlhF for polar formation, are required to inhibit minicell formation. Although the very initial steps in flagellar biosynthesis are unknown in *C. jejuni*, it is likely that the first components of a flagellum that are constructed include: FliF (which forms the inner membrane MS ring); FliG, FliM, and FliN (the motor/switch components of the cytoplasmic C ring); and the flagellar T3SS (which is located within the MS ring) [55]. These components mediate motor, switch, and secretory functions for a flagellum. Of note, the MS ring of *V. cholerae* appears to be dependent on FlhF for polar localization [5].

We analyzed a panel of *C. jejuni* mutants lacking these flagellar components for a defect in cell division that results in production of minicells. Inactivation of *fliF*, *fliM*, and *fliN* resulted in strong minicell phenotypes comparable to *C. jejuni* Δ*flhF* and Δ*flhG* mutants (Figure 6A and Figure S5). In contrast, a *fliG* mutant or mutants lacking a single component of the flagellar T3SS either did not produce a significant level of minicells or showed a significantly reduced minicell phenotype relative to *C. jejuni* Δ*flhG* (Figure 6A). These findings suggest that the MS ring and switch complex (made up of FliM and FliN) are involved in a mechanism to influence cell division in *C. jejuni*.

To verify that minicells are products of cell division in the *flhF*, *fliF*, *fliM*, and *fliN* mutants, minicell production was monitored in mutants after exposure to cephalexin. In each case, the minicell population was reduced in cephalexin-treated cells (Figure 6B). In the *fliF*, *fliM*, and *fliN* mutants, the minicell population was reduced over 50%. We next determined if the minicell phenotypes of the *fliF*, *fliM*, and *fliN* mutants are linked to FlhG, similar to what we observed with *C. jejuni* Δ*flhF*. When we overexpressed *flhG in trans* in each of these mutants, the minicell phenotype decreased (Figure 6C). Furthermore, deletion of *flhG* in the *fliF*, *fliM*, or *fliN* mutants did not augment minicell production in the mutants compared to single deletions of each gene (Figure 6D), suggesting that the MS and switch complex of flagella function in the same pathway as FlhG to influence a mechanism to inhibit cell division. Considering our findings, we surmise that polar flagellar biosynthesis influences formation of cell division sites via FlhG to ultimately result in symmetrical cell division in *C. jejuni*.

## Discussion

Elegant studies of *E. coli*, *B. subtilis*, and *C. crescentus* have elucidated highly refined mechanisms by which bacteria regulate precise placement of the divisome to promote cell division and generate viable progenitors. Although the mechanisms vary, a common theme in regulating divisome placement is that bacterial poles are usually protected from cell division so that the divisome more likely forms near the cellular midpoint for symmetrical division. We have identified a new collection of factors that compose a system to regulate formation of cell division sites in *C. jejuni*. This novel system is composed of the ParA ATPase family member FlhG and the MS ring and switch complex of polar flagella. Our work indicates that FlhG numerically controls amphitrichous flagellation and spatially regulates cell division. Furthermore, components of polar flagella are required for FlhG produced at normal levels to inhibit cell division specifically at polar regions. In the absence of FlhG, the MS ring, or switch components, cell division at poles more freely occurs to generate minicells. Thus, FlhG and polar flagellar biosynthesis block cell division from occurring at poles so that symmetrical division predominates to ensure generation of viable progenitors.

Other ParA ATPase family members, such as *E. coli* and *B. subtilis* MinD or *C. crescentus* MipZ, regulate formation of cell division sites by protecting poles from cell division, but the mechanisms by which these proteins function differ. MinD proteins do not directly inhibit cell division. Instead, these proteins localize MinC, the FtsZ inhibitor, to poles or maturing septa to inhibit divisome formation [10]–[13], [15], [56]. In contrast, *C. crescentus* MipZ directly mediates FtsZ depolymerization at polar sites [36]. *C. jejuni* FlhG is more homologous to MinDs than MipZ, with approximately 55% similarity and 35% identity between a 167-amino acid region of FlhG and *E. coli* MinD. This region includes the ATPase domains and some surrounding residues. In addition, a C-terminal amphipathic helix present in MinDs, but absent in MipZ, that is required for membrane interactions at poles to mediate inhibition of cell division is predicted at the C-terminus of FlhG [10], [57]–[59]. In contrast, FlhG and MipZ only share homology that is limited to a 40-amino acid region within the ATPase domains.

Considering how MinD and MipZ spatially mediate inhibition of cell division, it is currently unclear how FlhG may modulate cell division in *C. jejuni*. Production of FlhG_D61A_, which is predicted to be locked into an ATP-bound state, resulted in an elongated phenotype likely due to the mutant FlhG protein conferring a heightened cell division inhibitory activity. These results suggest that cycles of ATP binding and hydrolysis by FlhG are likely important for normal spatial regulation of Z-ring formation, similar to what has been observed with MinD and MipZ [18]–[20], [36], [60]. Due to the lack of genes encoding MinC and MinE in *C. jejuni*, it is unlikely that FlhG functions in an identical mechanism as MinD to inhibit cell division. However, FlhG may interact with and polarly localize other proteins with MinC-like functions in directly inhibiting Z-ring formation. Alternatively, FlhG may function similarly to *C. crescentus* MipZ to directly interact with FtsZ and inhibit Z-ring formation at poles. However, preliminary experiments failed to demonstrate that purified FlhG stimulated the GTPase activity of *C. jejuni* FtsZ *in vitro* (MB and DRH, unpublished observations), which would promote depolymerization of Z-rings into FtsZ monomers and inhibit cell division in the bacterium [61]–[63]. In addition, we were unable to detect an *in vitro* ATPase activity for FlhG (MB and DRH, unpublished observations), yet *in vivo* analysis of the elongated phenotype promoted by the *flhG_D61A_* mutant suggested that FlhG binds and hydrolyzes ATP for normal spatial regulation of Z-ring formation. These results indicate either that our *in vitro* conditions are not optimal for detecting a direct inhibitory activity of FlhG for FtsZ polymerization into Z-rings or that other components are required to activate or function with FlhG to inhibit FtsZ polymerization. Due to the requirement of flagellar components to limit cell division at poles, FlhG may require intact flagellar MS ring and switch structures to inhibit Z-ring formation and cell division.

As with the Min and MipZ division-site determination systems, we expect that an FlhG-dependent mechanism to protect poles from cell division likely requires some sort of topological specificity factor that either spatially confines or specifically activates this system at polar regions. Due to the minicell phenotype of MS ring and switch complex mutants, it is tempting to speculate that formation of a MS ring and C-ring switch complex at a pole, which is numerically regulated by FlhG, forms a topological specificity factor that assists FlhG and possibly other associated factors to facilitate inhibition of cell division specifically at poles. Without *fliF*, *fliM*, and *fliN*, a mechanism involving FlhG to inhibit cell division at poles is inoperable. Furthermore, the lack of an elongated cell phenotype in these mutants also suggests that this mechanism is not simply spatially deregulated and blocking divisome formation throughout the cell. Hence, specific flagellar components function with FlhG to inhibit cell division when the protein is produced at normal levels in the bacterium.

The minicell phenotype of *fliF*, *fliM*, and *fliN* mutants could be suppressed by increasing expression of *flhG*. Furthermore, analysis of double mutants lacking *flhG* and either *fliF*, *fliM*, or *fliN* did not reveal an increase in minicell production compared to the deletion of *flhG* alone. These findings together suggest that FlhG functions downstream of these flagellar components to influence cell division, rather than FlhG, the MS ring, and switch complex functioning in two separate pathways to influence cell division. Curiously, the base of the MS ring (composed by FliF) and the FliM and FliN structures in the switch complex of the C ring are cytoplasmic-accessible portions of the flagellar organelle, which may be available to interact with FlhG. In contrast, the flagellar T3SS and FliG, which are internal components of the MS and C rings, respectively, did not appear to be required to inhibit cell division at poles. If the MS ring and switch complex compose a topological specificity factor, FlhG may require a superficial domain of the flagellar motor and switch complex to initiate a mechanism to spatially inhibit cell division specifically at polar regions. Possible hypotheses for a mechanism by which flagellar components assist in modulating cell division include: 1) the flagellar structures may interact with FlhG to accumulate the protein to a critical concentration necessary to specifically inhibit Z-ring formation and cell division at poles; or 2) specific flagellar proteins or flagellum-associated components may activate a FlhG-dependent mechanism to inhibit cell division that is spatially confined to poles. Currently, our data do not allow for discerning which hypothesis may be true. The observation that FlhG alone did not stimulate the GTPase activity of *C. jejuni* FtsZ in preliminary assays suggests that FlhG may require intact flagellar components or other factors to promote FtsZ depolymerizaton (MB and DRH, unpublished observations). On the other hand, the fact that *flhG* overexpression suppressed the minicell phenotype of MS ring and switch complex mutants suggest that a FlhG-dependent mechanism to inhibit cell division is functional without flagella if FlhG levels are artificially high, which may add more strength to the hypothesis that polar flagellar structures promote polar accumulation of FlhG when produced at normal levels to inhibit cell division at poles. We attempted to determine if polar localization of FlhG-GFP was dependent on the flagellar motor and switch complex. Due to the low number of cells producing fluorescence and the low level of fluorescence of the fusion protein in these flagellar mutants, we were unable to confidently conclude that polar flagellar structures are required to localize FlhG to poles (MB and DRH, unpublished observations). Improved fluorescence microscopic procedures will be required to identify factors required for polar localization of FlhG. Although much more is to be learned about this system, our data suggest that polar flagellar biosynthesis functions with FlhG to inhibit cell division at poles.

Our findings have also revealed a previously unrecognized biological advantage for amphitrichous flagellation of *C. jejuni* that extends beyond an obvious role in promoting motility. Amphitrichous flagellation confers a characteristic darting motility for *C. jejuni* that assists in colonization of the intestinal mucosa in hosts [64], [65]. However, our studies have found that *C. jejuni* possesses a flagellum-influenced cell division inhibition system. The construction of such a system appears to allow amphitrichous flagellar biosynthesis, which is numerically controlled by FlhG, to influence an FlhG-dependent mechanism to prevent cell division at both poles. We propose that immediately after cell division, two daughter cells lack a flagellum at the new pole. Initiation of a single round of flagellar biosynthesis at this pole to complete the amphitrichous flagellation pattern would result in a MS ring and switch complex structure that an FlhG-dependent mechanism requires to inhibit cell division specifically at the new pole. A strictly monotrichous flagellar pattern in *C. jejuni* may inhibit cell division only at the flagellated pole with the aflagellated pole susceptible to divisome formation. A peritrichous flagellation pattern in *C. jejuni* may inhibit division throughout the cell. As such, amphitrichous flagella facilitate a mechanism so that FlhG-dependent cell division inhibition primarily occurs at the poles, which encourages symmetrical cell division to generate the highest number of viable *C. jejuni* daughter cells.

The polarly-flagellated bacteria commonly studied for motility, such as *Vibrio*, *Pseudomonas*, and *Helicobacter* species, encode FlhG and all Min components, except for *Campylobacter* species. A likely hypothesis for most polar flagellates is that FlhG controls numerical parameters of polar flagellar biosynthesis, whereas the Min system influences division-site placement. However, we observed that *H. pylori* FlhG, and to a lesser extent *V. cholerae* FlhG, resolved the minicell phenotype of *C. jejuni* Δ*flhG*, indicating that these proteins have the ability to influence cell division and possibly spatially regulate divisome formation. Therefore, the ability of FlhG to influence cell division may extend to other polarly-flagellated bacteria and form a broad mechanism used by many other motile bacteria to regulate cell division processes.

In this work, we have established a new paradigm that links polar flagellar biosynthesis to cell division in bacteria. Furthermore, we showed how amphitrichous flagellation is beneficial for influencing symmetrical cell division in *Campylobacter* so that two normal daughter cells are generated during each round of division. In addition, we provided a new function for the flagellar MS ring and switch complex in functioning with FlhG to prevent cell division from occurring at polar sites. Further exploration of this system will undoubtedly lead to a new understanding of the process of cell division that may occur in a broad range of polar flagellates.

## Materials and Methods

### Bacterial strains and growth conditions

All *C. jejuni* 81–176 Sm^R^ strains and procedures for generating mutants are described in Text S1 and Tables S1 and S2. For all experiments, *C. jejuni* strains were grown from freezer stocks on Mueller-Hinton (MH) agar containing 10 µg/ml trimethoprim for 48 h under microaerobic conditions at 37°C. Strains with plasmids for complementation analyses were grown with 50 µg/ml kanamycin. Strains were then restreaked onto MH agar containing appropriate antibiotics and grown for an additional 16 h and then used in experiments accordingly as described.

### Construction of plasmids for complementation, overexpression of genes, and analysis of polar localization of proteins

Introduction of *flhG* or *minD* alleles on plasmids into wild-type *C. jejuni* 81–176 Sm^R^ or *C. jejuni* 81–176 Sm^R^ Δ*flhG* for overexpression or complementation was accomplished by amplifying the alleles with primers containing 5' BamHI restriction sites immediately upstream of the start and stop codons of the respective genes. *flhG* alleles were amplified from the chromosomal DNA of *C. jejuni* 81–176, *H. pylori* J99, and *V. cholerae* O395. *minD* alleles were amplified from the chromosomal DNA of *H. pylori* J99, *V. cholerae* O395, and *E. coli* MG1655. The alleles were cloned into BamHI-digested pCE107, an *E. coli-C. jejuni* shuttle vector containing the σ^28^-dependent *flaA* promoter of *C. jejuni* followed by a BamHI site fused in-frame to a gene for the *Zoanthus* species green-fluorescent protein [66]. Insertion of *flhG* or *minD* alleles in the correct orientation placed a stop codon between the allele and the gene for GFP, preventing the formation of a fusion protein. All plasmids were sequenced and then transformed into DH5α/RK212.1 [67]. The plasmids were then conjugated into the appropriate *C. jejuni* 81–176 Sm^R^ strains by a previously published method [68].

To increase expression of *ftsZ* in *C. jejuni* Sm^R^ *flhG_D61A_* (MB1054), *ftsZ* was amplified from chromosomal DNA of *C. jejuni* 81–176 using primers containing 5' BamHI restriction sites immediately upstream of the start and stop codons of the gene. *ftsZ* was cloned into BamHI-digested pCE107 and then conjugated into wild-type *C. jejuni* or the *flhG_D61A_* mutant as described above. Expression of *ftsZ* from this plasmid, along with constitutive expression of *ftsZ* from the native chromosomal locus allowed for expression of *ftsZ* at increased levels relative to wild-type *C. jejuni*.

To increase expression of *flhG* in the *C. jejuni* Δ*flhF* (DRH1056), Δ*fliF* (DRH2074), *fliM* (DRH3304), and *fliN* (DRH1407) mutants, the coding sequence of *flhG* was amplified from chromosomal DNA from *C. jejuni* 81–176 with primers containing BamHI restriction sites in-frame to codon 2 and the stop codon. *flhG* was then cloned into BamHI-digested pECO101, an *E. coli*-*C. jejuni* shuttle vector containing the promoter of the chloramphenicol-acetyltransferase (*cat*) gene. After screening for correct orientation of *flhG* and sequencing, one plasmid (pMB1230) was transformed into DH5α/RK212.1 for conjugation into *C. jejuni* mutants as described above. Constitutive expression of *flhG* from the *cat* promoter on the plasmid, along with constitutive expression of *flhG* from the native chromosomal locus allowed for expression of *flhG* at increased levels relative to wild-type *C. jejuni*.

Construction of a plasmid to produce a FlhG-GFP fusion protein was accomplished by amplifying the coding sequence of *flhG* from *C. jejuni* 81–176 chromosomal DNA with primers containing BamHI restriction sites in frame the start and penultimate codons. This fragment was then cloned into BamHI-digested pCE107 [53]. One plasmid, pMB722, contained *flhG* in the correct orientation to produce a FlhG-GFP fusion protein. pMB722 and pCE107, which expressed GFP alone, was transformed into DH5α/pRK212.1 for conjugation into *C. jejuni* 81–176 Sm^R^ Δ*flhG* (MB770) as described above.

### Electron microscopy analysis

Strains were grown for 16 h on agar plates and then resuspended in PBS, pelleted for 3 min at full speed in a microcentrifuge, resuspended in 2% gluteraldehyde in 0.1 M cacodylate, and then incubated on ice for 1 h for fixation. Samples were then stained with 2% uranyl acetate and visualized with a FEI Technai G2 Spirit BioTWIN transmission electron microscope.

For analysis of the effect of cephalexin treatment on cell length and minicell production, strains were suspended from agar plates after growth for 16 h on agar plates and diluted to an OD_600_ 1.0. Each strain was diluted 1∶10 in MH broth in two separate flasks. Cephalexin was added at a final concentration of 15 or 12.5 µg/ml for wild-type *C. jejuni* or the *C. jejuni* Δ*flhG* mutant, respectively. Cultures were then incubated under microaerobic conditions at 37°C for 6 h. Samples were taken at time 0 h and 6 h to determine the number of viable bacteria in each culture and for analysis by electron microscopy. Samples were then fixed and stained as described above for visualization by transmission electron microscopy.

Data from two separate experiments were combined and averaged to determine the proportion of bacterial populations producing different numbers of polar flagella or cell bodies of different lengths as visualized by transmission electron microscopy. In total, over 210 individual bacteria were analyzed for each strain. For analysis of flagellar numbers, bacteria were placed into one of three categories: >2 flagella, producing at least two flagella at one or both poles; wild-type flagella, producing a single flagellum at one or both poles; or 0 flagella, aflagellated bacteria. For analysis of cell body lengths, bacteria were placed into one of four categories:<0.5 µm, minicells; 0.5–1 µm; 1–2 µm; and >2 µm. After averaging, the standard error for each population was calculated.

### Fluorescence microscopy analysis

After 16 h growth, bacteria were suspended from agar plates in MH broth and then diluted to OD_600_ 1.0. Approximately 1.5 ml of culture was pelleted in a microcentrifuge, followed by fixation with 4% formalin. Then, 350 µl of fixed cells were stained with 20 µl of 1 mg/ml FM4–64 for 15 min. Samples were added to poly-L-lysine-coated chamber slides. After 5 min, excess liquid was removed with a vacuum. ProLong Gold antifade reagent was applied to the chamber slides. After 24 h, fluorescent images were obtained with an Applied Precision PersonalDV deconvolution microscope with an Olympus 100x objective lens and a CoolSNAP_HQ2 camera. Images were processed using the ImageJ program.

### Accession numbers

The following GenBank accession numbers identify all previously uncharacterized proteins that were analyzed in this work: *Campylobacter jejuni* 81–176 FlhG, EAQ71939; *Campylobacter jejuni* 81–176 FliE, EAQ73125; *Campylobacter jejuni* 81–176 FliQ, EAQ72806; *Campylobacter jejuni* 81–176 FliM, EAQ71948; *Campylobacter jejuni* 81–176 FliN, 73197; *Campylobacter jejuni* 81–176 FliG, EAQ73253; *Helicobacter pylori* 26695 FlhG, AAD08077; *Helicobacter pylori* 26695 MinD, AAD07400; *Vibrio cholerae* O395 MinD, ACP10067.

## Supporting Information

Figure S1 **Alignment of FlhG and MinD proteins from different bacteria.** ClustalW alignment of the amino acid sequence of the FlhG proteins from *C. jejuni* 81–176, *H. pylori* 26695, and *V. cholerae* O395 and MinD proteins from *H. pylori* 26695, *V. cholerae* O395, and *E. coli* K12 substrain MG1655. The predicted ATPase domain of each protein common to the ParA ATPase family of proteins is outlined in blue, with the deviant Walker A motif common to family members outlined in black. The conserved aspartic acid residue of the ParA ATPAse family members proposed to be required for ATP hydrolysis is indicated in red. This residue corresponds to D61 in *C. jejuni* FlhG, which was mutated in this study. Conserved residues are indicated with an asterisk (*); highly conserved residues are indicated by a colon (:); and semi-conserved residues are indicated by a dot (.). GenBank accession numbers for proteins included for analysis are: *C. jejuni* 81–176 FlhG (CjFlgF; EAQ71939); *H. pylori* 26695 FlhG (HpFlhG; AAD08077); *V. cholerae* O395 FlhG (VcFlhG; ACP10174); *H. pylori* 26695 MinD (HpMinD; AAD07400); *V. cholerae* O396 MinD (VcMinD; ACP10067) and *E. coli* K-12 substrain MG1655 (EcMinD; AAC74259)
(TIF)

Figure S2 **Effect on** ***flhG*** **mutation on motility and flagellar gene expression.** (A) Motility phenotype of wild-type *C. jejuni* and Δ*flhG* mutant strains in semi-solid agar. Cultures of similar densities were stabbed into motility agar and incubated in microaerobic conditions at 37°C for 24 h. The *C. jejuni* Δ*flhG* mutant was complemented with empty vector or plasmid expressing *flhG*. (B) Arylsulfatase assays measuring the level of flagellar gene expression in wild-type *C. jejuni* and Δ*flhG* mutant strains. Transcriptional fusions of flagellar genes linked to a promoterless *astA* gene were used to replace respective wild-type alleles in *C. jejuni* Δ*astA* (wild-type *C. jejuni*; blue bars) or *C. jejuni* Δ*astA* Δ*flhG* (red bars). Results are from a typical assay with each strain performed in triplicate. Values reported for each strain are average arylsulfatase activity ± standard deviation relative to the amount of expression of each transcriptional fusion in wild-type *C. jejuni* Δ*astA*. Genes analyzed include those for the flagellar type III secretion system (early class of flagellar genes), σ^54^-dependent middle class of flagellar genes, and σ^28^-dependent late class of flagella genes.
(TIF)

Figure S3 **Distribution of cell lengths in populations of wild-type** ***C. jejuni*** **and** ***C. jejuni*** **Δ** ***flhG*** **.** The length of the cell bodies of wild-type *C. jejuni* 81–176 and *C. jejuni* Δ*flhG* populations were measured. Approximately 300 individual bacteria in each population were analyzed. Bacteria were divided into groups with lengths that ranged between 0.1 µm increments. The number of bacteria in each group is reported as a percentage of the entire bacterial population. Blue bars and red bars indicate the distribution of wild-type *C. jejuni* and *C. jejuni* Δ*flhG*, respectively.
(TIF)

Figure S4 **Distribution of cell lengths in populations of wild-type** ***C. jejuni*** **and** ***C. jejuni flhG_D61A_*** **.** The length of the cell bodies of wild-type *C. jejuni* 81–176 and *C. jejuni flhG_D61A_* populations were measured. Approximately 300 individual bacteria in each population were analyzed. Bacteria between 0.1 and 4.0 µm were divided into groups with lengths that ranged between 0.1 µm increments. Bacteria with lengths above 4.0 µm were divided into groups with lengths that ranged between 1.0 µm. The number of bacteria in each group is reported as a percentage of the entire bacterial population. Blue bars and red bars indicate the distribution of wild-type *C. jejuni* and *C. jejuni flhG_D61A_*, respectively.
(TIF)

Figure S5 **Minicell production in** ***C. jejuni flhF*** **,** ***fliF*** **,** ***fliM*** **, and** ***fliN*** **mutants.** Electron micrographs of negatively-stained *C. jejuni* mutants and associated minicells. Red arrows indicate minicells being generated at the pole of a bacterium or alongside bacteria of normal lengths. Bars = 1 µm.
(TIF)

Text S1 **Additional materials and methods.**
(DOC)

Table S1 **Bacterial strains used in this study.**
(DOC)

Table S2 **Plasmids used in this study.**
(DOC)
