# Supplementary figures and images for: Polar Flagellar Biosynthesis and a Regulator of Flagellar Number Influence Spatial Parameters of Cell Division in Campylobacter jejuni
Source: PLoS Pathog. 2011 Dec 1;7(12):e1002420. doi: 10.1371/journal.ppat.1002420 (PMC3228812; doi:10.1371/journal.ppat.1002420)

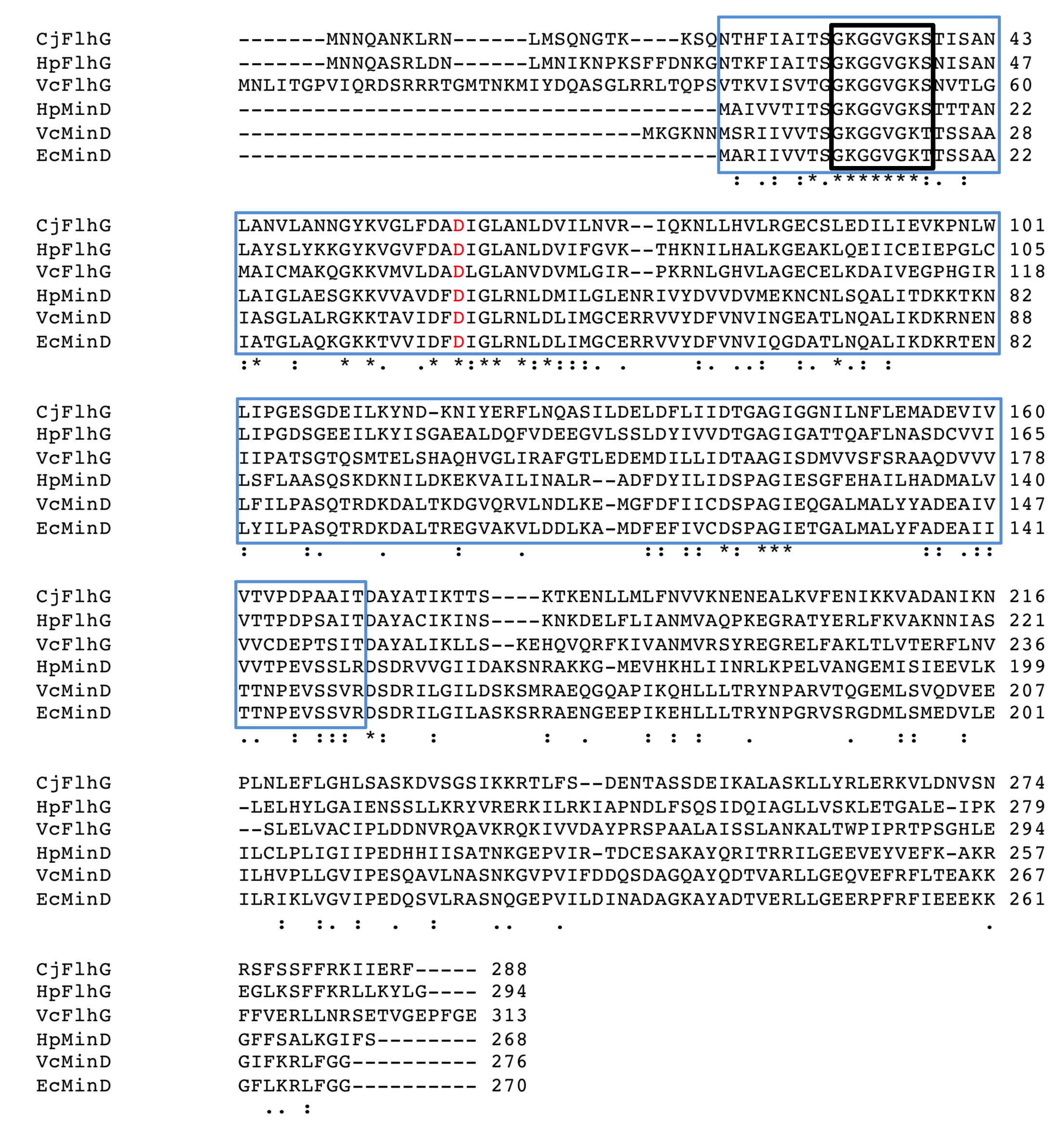

Supplement: Figure S1 — Alignment of FlhG and MinD proteins from different bacteria. ClustalW alignment of the amino acid sequence of the FlhG proteins from C. jejuni 81–176, H. pylori 26695, and V. cholerae O395 and MinD proteins from H. pylori 26695, V. cholerae O395, and E. coli K12 substrain MG1655. The predicted ATPase domain of each protein common to the ParA ATPase family of proteins is outlined in blue, with the deviant Walker A motif common to family members outlined in black. The conserved aspartic acid residue of the ParA ATPAse family members proposed to be required for ATP hydrolysis is indicated in red. This residue corresponds to D61 in C. jejuni FlhG, which was mutated in this study. Conserved residues are indicated with an asterisk (*); highly conserved residues are indicated by a colon (:); and semi-conserved residues are indicated by a dot (.). GenBank accession numbers for proteins included for analysis are: C. jejuni 81–176 FlhG (CjFlgF; EAQ71939); H. pylori 26695 FlhG (HpFlhG; AAD08077); V. cholerae O395 FlhG (VcFlhG; ACP10174); H. pylori 26695 MinD (HpMinD; AAD07400); V. cholerae O396 MinD (VcMinD; ACP10067) and E. coli K-12 substrain MG1655 (EcMinD; AAC74259) (TIF) [file ppat.1002420.s001.tif]

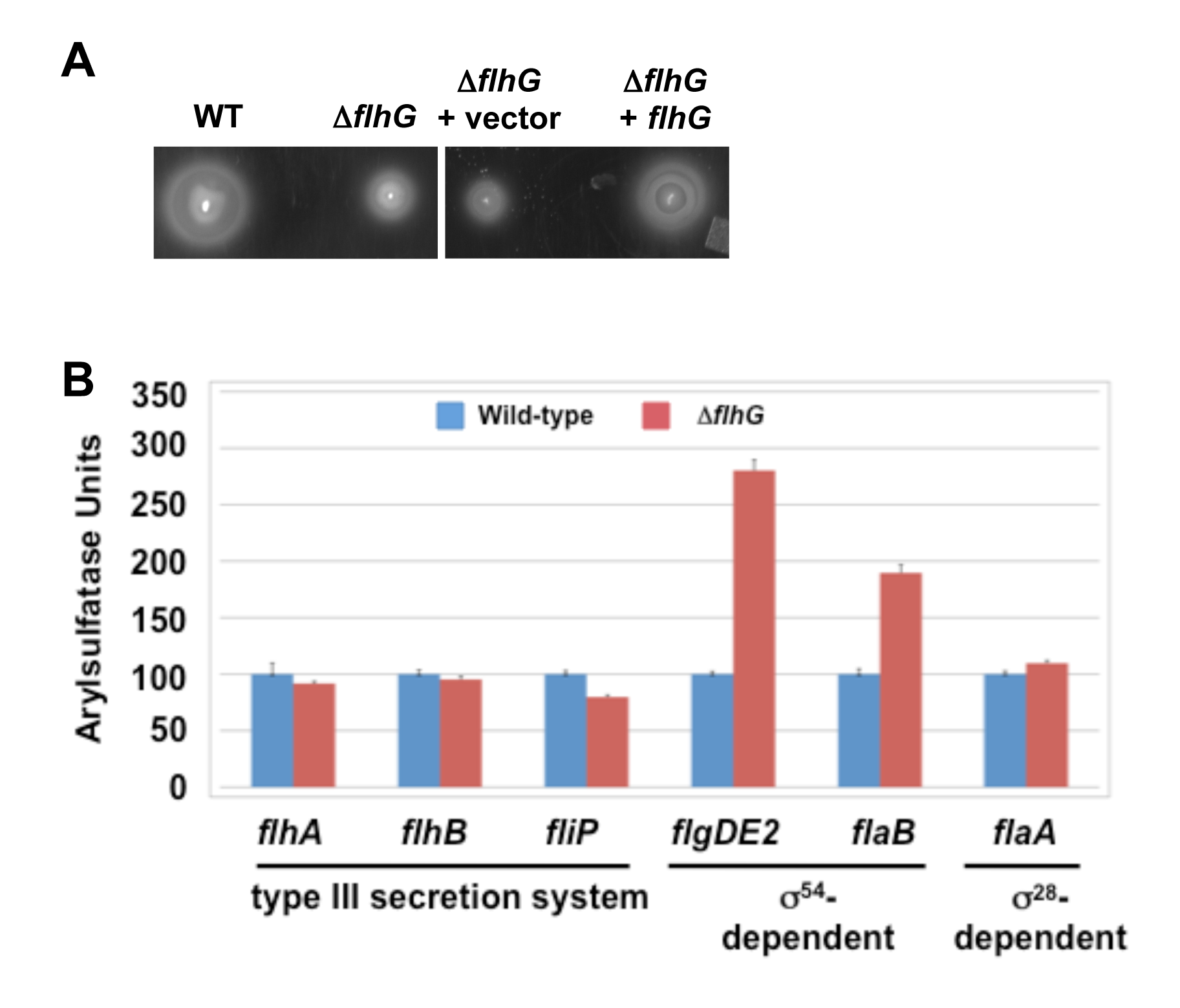

Supplement: Figure S2 — Effect on flhG mutation on motility and flagellar gene expression. (A) Motility phenotype of wild-type C. jejuni and ΔflhG mutant strains in semi-solid agar. Cultures of similar densities were stabbed into motility agar and incubated in microaerobic conditions at 37°C for 24 h. The C. jejuni ΔflhG mutant was complemented with empty vector or plasmid expressing flhG. (B) Arylsulfatase assays measuring the level of flagellar gene expression in wild-type C. jejuni and ΔflhG mutant strains. Transcriptional fusions of flagellar genes linked to a promoterless astA gene were used to replace respective wild-type alleles in C. jejuni ΔastA (wild-type C. jejuni; blue bars) or C. jejuni ΔastA ΔflhG (red bars). Results are from a typical assay with each strain performed in triplicate. Values reported for each strain are average arylsulfatase activity ± standard deviation relative to the amount of expression of each transcriptional fusion in wild-type C. jejuni ΔastA. Genes analyzed include those for the flagellar type III secretion system (early class of flagellar genes), σ54-dependent middle class of flagellar genes, and σ28-dependent late class of flagella genes. (TIF) [file ppat.1002420.s002.tif]

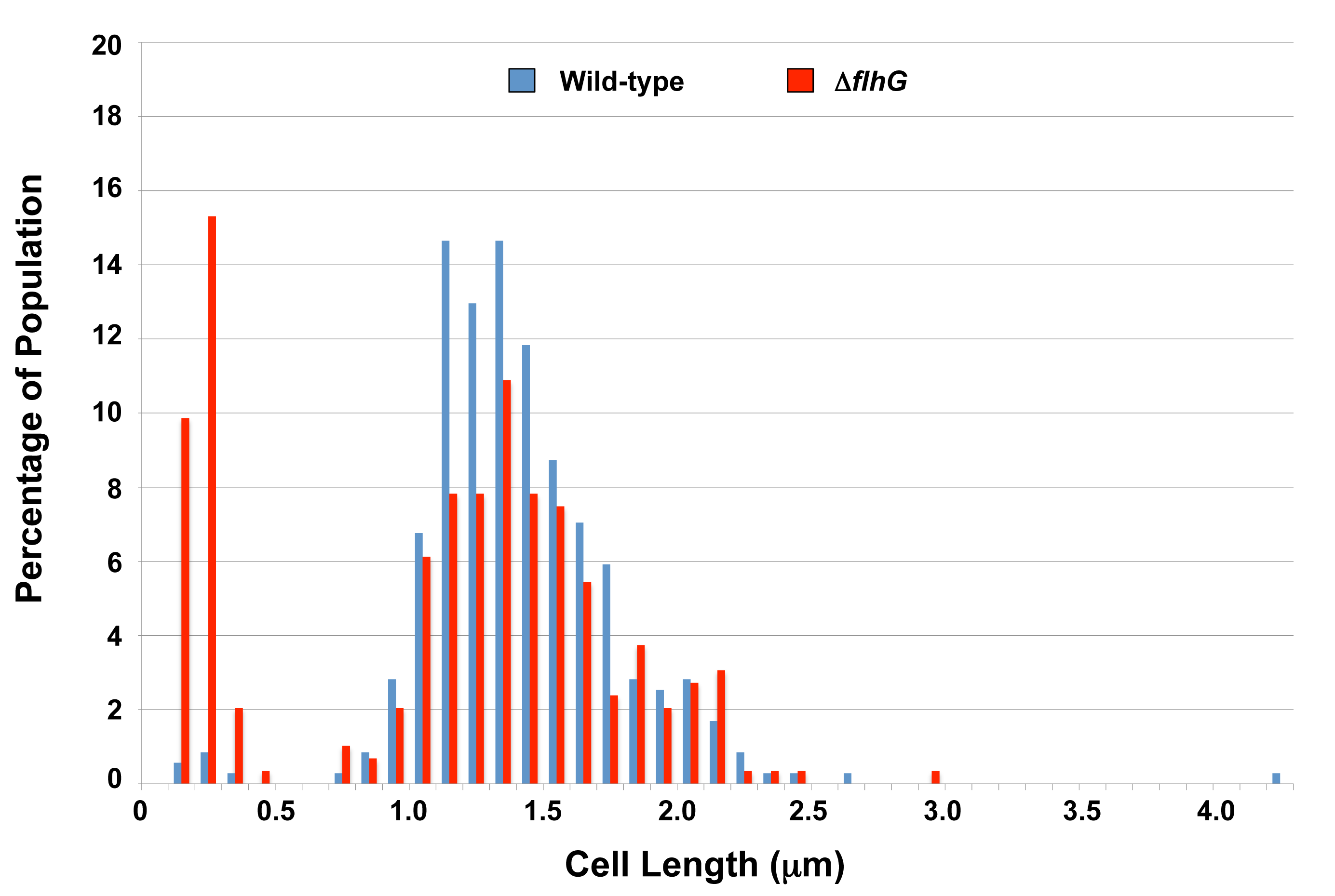

Supplement: Figure S3 — Distribution of cell lengths in populations of wild-type C. jejuni and C. jejuni Δ flhG . The length of the cell bodies of wild-type C. jejuni 81–176 and C. jejuni ΔflhG populations were measured. Approximately 300 individual bacteria in each population were analyzed. Bacteria were divided into groups with lengths that ranged between 0.1 µm increments. The number of bacteria in each group is reported as a percentage of the entire bacterial population. Blue bars and red bars indicate the distribution of wild-type C. jejuni and C. jejuni ΔflhG, respectively. (TIF) [file ppat.1002420.s003.tif]

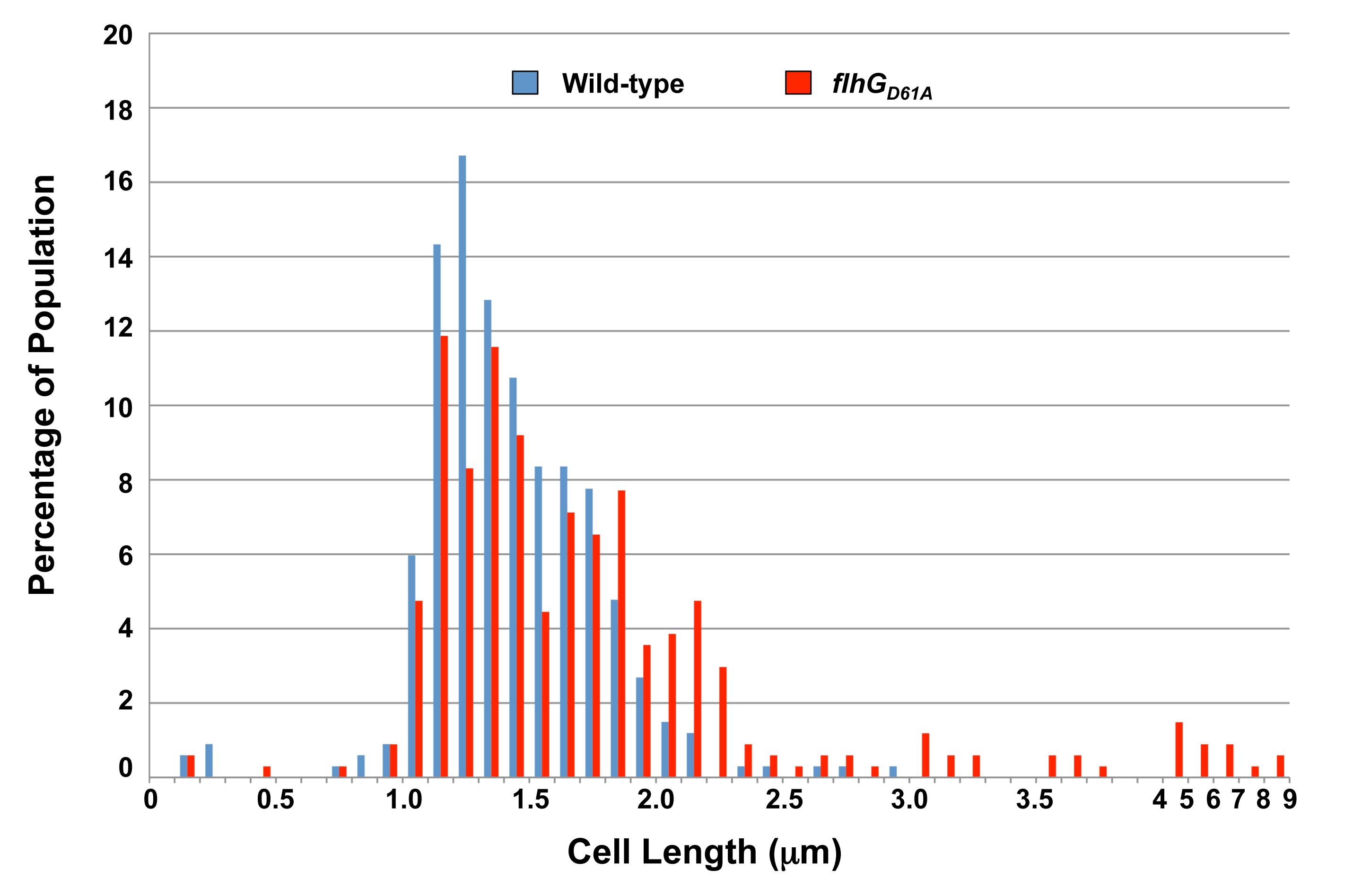

Supplement: Figure S4 — Distribution of cell lengths in populations of wild-type C. jejuni and C. jejuni flhGD61A . The length of the cell bodies of wild-type C. jejuni 81–176 and C. jejuni flhGD61A populations were measured. Approximately 300 individual bacteria in each population were analyzed. Bacteria between 0.1 and 4.0 µm were divided into groups with lengths that ranged between 0.1 µm increments. Bacteria with lengths above 4.0 µm were divided into groups with lengths that ranged between 1.0 µm. The number of bacteria in each group is reported as a percentage of the entire bacterial population. Blue bars and red bars indicate the distribution of wild-type C. jejuni and C. jejuni flhGD61A, respectively. (TIF) [file ppat.1002420.s004.tif]

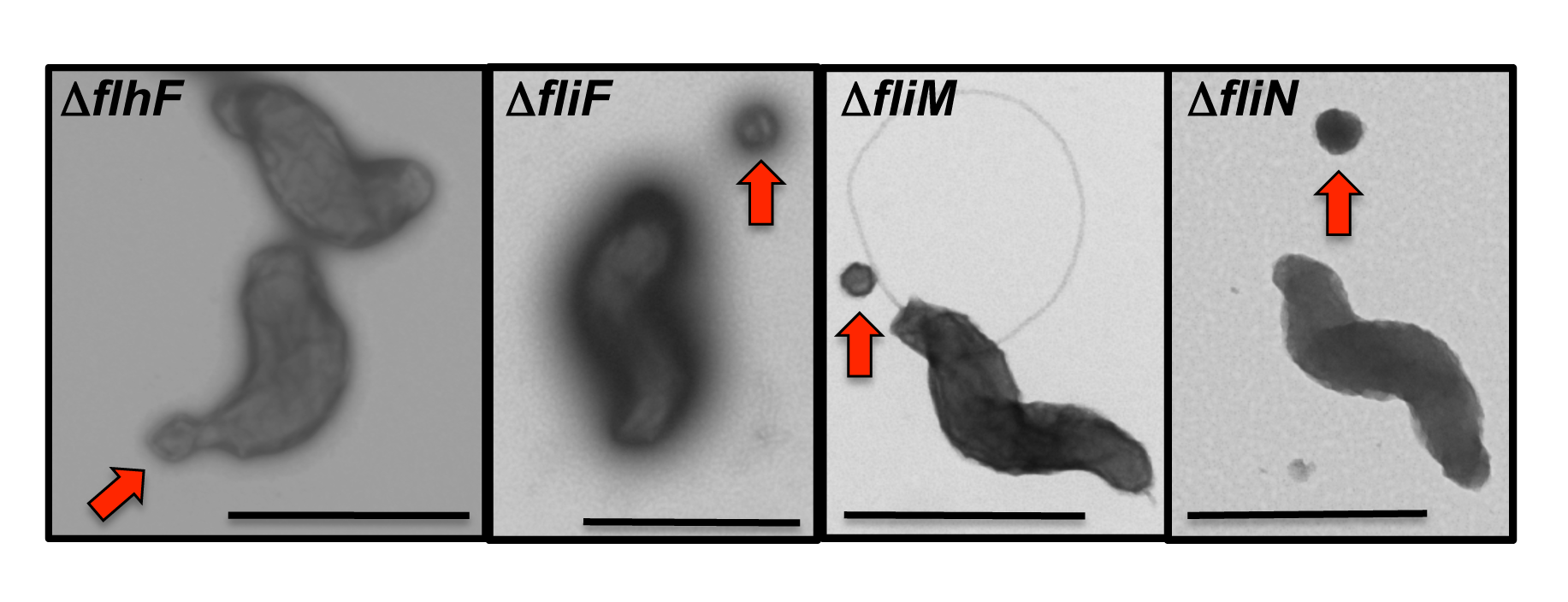

Supplement: Figure S5 — Minicell production in C. jejuni flhF , fliF , fliM , and fliN mutants. Electron micrographs of negatively-stained C. jejuni mutants and associated minicells. Red arrows indicate minicells being generated at the pole of a bacterium or alongside bacteria of normal lengths. Bars = 1 µm. (TIF) [file ppat.1002420.s005.tif]
