# Supplementary material for: Polar Flagellar Biosynthesis and a Regulator of Flagellar Number Influence Spatial Parameters of Cell Division in Campylobacter jejuni
Source: PLoS Pathog. 2011 Dec 1;7(12):e1002420. doi: 10.1371/journal.ppat.1002420 (PMC3228812; doi:10.1371/journal.ppat.1002420)
Supplement: Text S1 — Additional materials and methods. (DOC) [file ppat.1002420.s006.doc]

**Text S1. Additional materials and methods**

**Construction of mutant strains.**

*C. jejuni* 81-176 *rpsL*^Sm^ mutant strains previously described include: ∆*flhF* (DRH1056; [1]); ∆*flhA* (DRH946; [1]); ∆*flhB* (SNJ471; [2]); ∆*fliP* (DRH1065; [1]); ∆*fliR* (DRH755; [1]), ∆*rpoN* (DRH321; [3]), ∆*astA* (DRH461; [1]), and ∆*fliF* (DRH2074; [4]). *E. coli* DH5α was used for all cloning procedures. Electroporation of *C. jejuni* and creation of insertional and in-frame deletions were performed by previously published protocols [3,5].

***C. jejuni*** ∆***flhG* and *C. jejuni flhG_D61A_*.** *flhG* was amplified with 700 bp of flanking sequence by PCR from the *C. jejuni* 81-176 chromosomal DNA with primers containing 5’ BamHI sites. After cloning into BamHI-digested pUC19 (creating pSMS248), a MscI restriction site was introduced into the coding sequence of *flhG* using PCR-mediated mutagenesis (creating pSMS259). In addition, PCR-mediated mutagenesis was performed with pSMS248 to change the codon for D61 to a codon for alanine (creating pMB951 containing the *flhG_D61A_* allele) and to delete in-frame codons 14 - 241 (creating pMB752 containing the ∆*flhG* allele). A SmaI-digested *cat-rspL* cassette (from pDRH265; [3]) was inserted into the MscI site within *flhG* to create pSMS275 and pSMS279. *C. jejuni* 81-176 Sm^R^ (DRH212; [3]) was electroporated with pSMS275 and transformants were recovered on MH agar containing 10 μg/ml chloramphenicol. Two *C. jejuni* 81-176 Sm^R^ *flhG*::*cat-rspL* transformants (SMS368 and SMS370) were verified by PCR.

*C. jejuni* 81-176 Sm^R^ ∆*flhG* was created by electroporating SMS370 with pMB752. Transformants were recovered on MH agar containing 0.5 – 2 mg/ml streptomycin. Deletion of a major portion of *flhG* in one transformant (MB770) was verified by PCR. *C. jejuni* 81-176 Sm^R^ *flhG_D61A_* was created by electroporating SMS368 with pMB951. Transformants were recovered on MH agar containing 0.5 – 2 mg/ml streptomycin. The presence of *flhG_D61A_* at the native location on the chromosome in two transformants (MB1040 and MB1054) was verified by PCR and sequencing.

***C. jejuni*** ∆***astA*** ∆***flhG*.** *C. jejuni* 81-176 Sm^R^ ∆*astA* (DRH461; [1]) was electroporated with pSMS279 and transformants were recovered on MH agar containing 10 μg/ml chloramphenicol. One *C. jejuni* 81-176 Sm^R^ ∆*astA* *flhG*::*cat-rpsL* transformant (DRH2133) was verified by PCR. To replace *flhG*::*cat-rpsL* with the ∆*flhG* allele, DRH2133 was electroporated with pMB752. Transformants were recovered on MH agar containing 0.5 – 2 mg/ml streptomycin. Deletion of a major portion of *flhG* in one transformant (MB771) was verified by PCR.

***C. jejuni fliG*::*cat-rpsL*.** *fliG* was amplified with 700 bp of flanking sequence by PCR from *C. jejuni* 81-176 chromosomal DNA with primers containing 5’ BamHI sites. After cloning into BamHI-digested pUC19 (creating pDRH2407), a SmaI-digested *cat-rspL* cassette (from pDRH265) was inserted into the ClaI restriction site within *fliG* to create pALU115. *C. jejuni* 81-176 Sm^R^ (DRH212) was electroporated with pALU115 and transformants were recovered on MH agar containing 10 μg/ml chloramphenicol. One *C. jejuni* 81-176 Sm^R^ *fliG*::*cat-rspL* transformant (DRH2469) was verified by PCR.

***C. jejuni*** ∆***flhG fliF::cat-rpsL*.** *C. jejuni* 81-176 Sm^R^ ∆*flhG* was electroporated with pDRH1814 containing *fliF*::*cat-rpsL* [4]. Transformants were recovered on MH agar containing 10 μg/ml chloramphenicol. One *C. jejuni* 81-176 Sm^R^ ∆*flhG* *fliF*::*cat-rspL* transformant (MB806) was verified by PCR.

***C. jejuni fliM*::*cat-rpsL* and *C. jejuni*** ∆***flhG fliM::cat-rpsL*.** *fliM* was amplified with 700 bp of flanking sequence by PCR from *C. jejuni* 81-176 chromosomal DNA with primers containing 5’ BamHI sites. After cloning into BamHI-digested pUC19 (creating pJMB532), a SmaI-digested *cat-rspL* cassette (from pDRH265) was inserted into the EcoRV restriction site within *fliM* to create pJMB572. *C. jejuni* 81-176 Sm^R^ (DRH212) and 81-176 Sm^R^ ∆*flhG* (MB770) were electroporated with pJMB572 and transformants were recovered on MH agar containing 10 μg/ml chloramphenicol. One *C. jejuni* 81-176 Sm^R^ *fliM*::*cat-rspL* transformant (DRH3304) and one ∆*flhG* *fliM*::*cat-rpsL* transformant (DRH3363) were verified by PCR.

***C. jejuni fliN*::*cat-rpsL* and *C. jejuni*** ∆***flhG fliM::cat-rpsL*.** *fliN* was amplified with 1 kb of flanking sequence by PCR from *C. jejuni* 81-176 chromosomal DNA with primers containing 5’ BamHI sites. After cloning into BamHI-digested pUC19 (creating pDRH1350), a SmaI-digested *cat-rspL* cassette (from pDRH265) was inserted into the EcoRV restriction site within *fliN* to create pDRH1367. *C. jejuni* 81-176 Sm^R^ (DRH212) and 81-176 Sm^R^ ∆*flhG* (MB770) were electroporated with pDRH1367 and transformants were recovered on MH agar containing 10 μg/ml chloramphenicol. One *C. jejuni* 81-176 Sm^R^ *fliN*::*cat-rspL* transformant (DRH1407) and one ∆*flhG* *fliN*::*cat-rpsL* transformant (DRH3367) were verified by PCR.

***C. jejuni*** ∆***fliQ*.** *fliQ* was amplified with 700 bp of flanking sequence by PCR from *C. jejuni* 81-176 chromosomal DNA with primers containing 5’ BamHI sites. After cloning into BamHI-digested pUC19 (creating pDRH1454), a MscI restriction site was introduced into the coding sequence of *fliQ* using PCR-mediated mutagenesis (creating pSMS462). In addition, PCR-mediated mutagenesis was performed with pDRH1454 to delete in-frame codons 10-72 (creating pSMS443 containing the ∆*fliQ* allele). A SmaI-digested *cat-rspL* cassette (from pDRH265) was inserted into the MscI site within *fliQ* to create pSMS469. *C. jejuni* 81-176 Sm^R^ (DRH212) was electroporated with pSMS469 and transformants were recovered on MH agar containing 10 μg/ml chloramphenicol. One *C. jejuni* 81-176 Sm^R^ *fliQ*::*cat-rspL* transformant (SMS508) was verified by PCR.

*C. jejuni* 81-176 Sm^R^ ∆*fliQ* was created by electroporating SMS508 with pSMS443. Transformants were recovered on MH agar containing 0.5 – 2 mg/ml streptomycin. Deletion of a major portion of *fliQ* in one transformant (DAR101) was verified by PCR.

***C. jejuni*** ∆***fliE*.** The *flgBCfliE* locus was amplified with 600 to 700 bp of flanking sequence by PCR from the *C. jejuni* 81-176 chromosomal DNA with primers containing 5’ BamHI sites. After cloning into BamHI-digested pUC19 (creating pDRH2428), a StuI restriction site was introduced into the coding sequence of *fliE* using PCR-mediated mutagenesis (creating pSNJ822). In addition, PCR-mediated mutagenesis was performed with pDRH2428 to fuse in-frame the start codon to the last 20 codons of *fliE* (which results in deletion of codons 2 through 78) to create pSNJ918 containing the ∆*fliE* allele. A SmaI-digested *cat-rspL* cassette (from pDRH265) was inserted into the StuI site within *fliE* to create pSNJ878. *C. jejuni* 81-176 Sm^R^ ∆*astA* (DRH461) was electroporated with pSNJ878 and transformants were recovered on MH agar containing 10 μg/ml chloramphenicol. One *C. jejuni* 81-176 Sm^R^ ∆*astA* *fliE*::*cat-rspL* transformant (SNJ907) was verified by PCR.

*C. jejuni* 81-176 Sm^R^ ∆*astA* ∆*fliE* was created by electroporating SNJ907 with pSNJ918. Transformants were recovered on MH agar containing 0.5 – 2 mg/ml streptomycin. Deletion of a major portion of *fliE* in one transformant (SNJ915) was verified by PCR.

***astA* transcriptional reporter assays**

Transcriptional reporter gene fusions were constructed by inserting the SmaI *astA*-*kan* cassette from pDRH580 into the NcoI site of *flhA* in pDRH664 (to generate pDRH867 and create *flhA*::*astA-kan*), the MscI site of *fliP* in pSNJ128 (to generate pMB109 and create *fliP*::*astA-kan*), and the StuI site of *flhB* of pDRH742 (to generate pMB144 and create *flhB*::*astA-kan*).

*C. jejuni* strains in the ∆*astA* background were electroporated with pDRH532, pDRH608, pDRH610, pDRH867, pMB109, and pMB144 to create promoterless *astA* transcriptional fusions to *flgDE2*, *flaA*, *flaB*, *flhA*, *fliP*, and *flhB* respectively. Arylsulfatase production from the transcriptional fusions in these strains was measured by previously published methods [1,6,7].

**Motility assays**

Motility phenotypes of wild-type *C. jejuni* and mutant strains were assessed as previously described [2]. Briefly, strains were grown from freezer stocks on MH agar containing 10 μg/ml trimethoprim or 10 μg/ml chloramphenicol for 48 h at 37 °C in microaerobic conditions. Strains were restreaked on MH agar and grown for an additional 16 h. Strains were suspended from agar plates in MH broth to OD_600_ of 0.8 and stabbed into semisolid MH motility agar containing 10 μg/ml trimethoprim or 10 μg/ml chloramphenicol by using a sterilized inoculating needle. The plates were incubated for 24 h at 37 °C in microaerobic conditions and then visualized for motility.

**References**

1. Hendrixson DR, DiRita VJ (2003) Transcription of σ^54^-dependent but not σ^28^-dependent flagellar genes in *Campylobacter jejuni* is associated with formation of the flagellar secretory apparatus. Mol Microbiol 50: 687-702.

2. Joslin SN, Hendrixson DR (2009) Activation of the *Campylobacter jejuni* FlgSR two-component system is linked to the flagellar export apparatus. J Bacteriol 191: 2656-2667.

3. Hendrixson DR, Akerley BJ, DiRita VJ (2001) Transposon mutagenesis of *Campylobacter jejuni* identifies a bipartite energy taxis system required for motility. Mol Microbiol 40: 214-224.

4. Balaban M, Joslin SN, Hendrixson DR (2009) FlhF and its GTPase activity are required for distinct processes in flagellar gene regulation and biosynthesis in *Campylobacter jejuni*. J Bacteriol 191: 6602-6611.

5. Van Vliet AHM, Wood AC, Henderson J, Wooldridge K, Ketley JM (1997) Genetic manipulation of enteric *Campylobacter* species. Methods Microbiol 27: 407-419.

6. Henderson MJ, Milazzo FH (1979) Arylsulfatase in *Salmonella typhimurium*: detection and influence of carbon source and tyramine on its synthesis. J Bacteriol 139: 80-87.

7. Yao R, Guerry P (1996) Molecular cloning and site-specific mutagenesis of a gene involved in arylsulfatase production in *Campylobacter jejuni*. J Bacteriol 178: 3335-3338.

8. Figurski DH, Helinski DR (1979) Replication of an origin-containing derivative of plasmid RK2 dependent on a plasmid function provided in trans. Proc Natl Acad Sci U S A 76: 1648-1652.

9. Blattner FR, Plunkett G, 3rd, Bloch CA, Perna NT, Burland V, et al. (1997) The complete genome sequence of *Escherichia coli* K-12. Science 277: 1453-1462.

10. Mekalanos JJ, Swartz DJ, Pearson GD, Harford N, Groyne F, et al. (1983) Cholera toxin genes: nucleotide sequence, deletion analysis and vaccine development. Nature 306: 551-557.

11. Alm RA, Ling LS, Moir DT, King BL, Brown ED, et al. (1999) Genomic-sequence comparison of two unrelated isolates of the human gastric pathogen *Helicobacter pylori*. Nature 397: 176-180.

12. Korlath JA, Osterholm MT, Judy LA, Forfang JC, Robinson RA (1985) A point-source outbreak of campylobacteriosis associated with consumption of raw milk. J Infect Dis 152: 592-596.

13. Yao R, Alm RA, Trust TJ, Guerry P (1993) Construction of new *Campylobacter* cloning vectors and a new mutational *cat* cassette. Gene 130: 127-130.

14. Elliott KT, Dirita VJ (2008) Characterization of CetA and CetB, a bipartite energy taxis system in *Campylobacter jejuni*. Mol Microbiol 69: 1091-1103.

15. Ewing CP, Andreishcheva E, Guerry P (2009) Functional characterization of flagellin glycosylation in *Campylobacter jejuni* 81-176. J Bacteriol 191: 7086-7093.
