# Supplementary material for: Polar Flagellar Biosynthesis and a Regulator of Flagellar Number Influence Spatial Parameters of Cell Division in Campylobacter jejuni
Source: PLoS Pathog. 2011 Dec 1;7(12):e1002420. doi: 10.1371/journal.ppat.1002420 (PMC3228812; doi:10.1371/journal.ppat.1002420)
Supplement: Table S1 — Bacterial strains used in this study. (DOC) [file ppat.1002420.s007.doc]

Table S1. Bacterial strains used in this study

| **Strain** | **Genotype** | **Source/Reference** |
| --- | --- | --- |
| DH5α | *E. coli supE44* ∆*lac*U169 (φ80*lacZ*ΔM15) *hsdR*17 *recA*1 *endA*1 *gyrA*96 *thi*-1 *relA*1 | Invitrogen |
| DH5α/RK212.1 | DH5α with conjugation transfer element | [8] |
| MG1655 | *E. coli* | [9] |
| O395 | *V. cholerae* | [10] |
| J99 | *H. pylori* | [11] |
| 81-176 | wild-type *C. jejuni* clinical isolate | [12] |
| DRH212 | 81-176 *rpsL^Sm^* | [3] |
| DRH321 | DRH212 ∆*rpoN* | [3] |
| DRH461 | DRH212 ∆*astA* | [1] |
| DRH533 | DRH461 *flgDE2*::*nemo* | [1] |
| DRH655 | DRH461 *flaA*::*astA-kan* | [1] |
| DRH665 | DRH461 *flaB*::*astA-kan* | [1] |
| DRH755 | DRH212 ∆*fliR* | [1] |
| DRH946 | DRH212 ∆*flhA* | [1] |
| DRH1056 | DRH212 ∆*flhF* | [1] |
| DRH1065 | DRH212 ∆*fliP* | [1] |
| DRH1407 | DRH212 *fliN*::*cat-rpsL* | This study |
| DRH2074 | DRH212 ∆*fliF* | [4] |
| DRH2133 | DRH461 *flhG*::*cat-rpsL* | This study |
| DRH2204 | DRH461 ∆*flhG* | This study |
| DRH2469 | DRH212 *fliG*::*cat-rpsL* | This study |
| DRH3304 | DRH212 *fliM*::*cat-rpsL* | This study |
| DRH3363 | DRH212 ∆*flhG* *fliM*::*cat-rpsL* | This study |
| DRH3367 | DRH212 ∆*flhG* *fliN*::*cat-rpsL* | This study |
| DRH3368 | MB770/pMB722 | This study |
| DAR101 | DRH212 ∆*fliQ* | This study |
| SMS368 | DRH212 *flhG*::*cat-rpsL* | This study |
| SMS370 | DRH212 *flhG*::*cat-rpsL* | This study |
| SMS508 | DRH212 *fliQ*::*cat-rpsL* | This study |
| MB117 | DRH461 *flhA*::*astA-kan* | This study |
| MB150 | DRH461 *fliP*::*astA-kan* | This study |
| MB164 | DRH461 *flhB*::*astA-kan* | This study |
| MB269 | DRH2204 *fliP*::*astA-kan* | This study |
| MB341 | DRH2204 *flhB*::*astA-kan* | This study |
| MB344 | DRH2204 *flhA*::*astA-kan* | This study |
| MB770 | DRH212 ∆*flhG* | This study |
| MB771 | DRH461 ∆*flhG* | This study |
| MB806 | DRH212 ∆*flhG* *fliF*::*cat-rpsL* | This study |
| MB847 | MB771 *flaA*::*astA-kan* | This study |
| MB849 | MB771 *flaB*::*astA-kan* | This study |
| MB859 | MB771 *flgE2*::*nemo* | This study |
| MB970 | MB770/pMB865 | This study |
| MB972 | MB770/pCE107 | This study |
| MB975 | MB770/pMB913 | This study |
| MB977 | MB770/pMB915 | This study |
| MB981 | MB770/pMB917 | This study |
| MB1040 | DRH212 *flhG_D61A_* | This study |
| MB1054 | DRH212 *flhG_D61A_* | This study |
| MB1069 | MB770/pMB1014 | This study |
| MB1101 | MB770/pMB1018 | This study |
| MB1176 | MB1054/pMB1142 | This study |
| MB1209 | DRH1065/pRY108 | This study |
| MB1241 | DRH1065/pMB1230 | This study |
| MB1248 | DRH2074/pMB1230 | This study |
| MB1253 | DRH3304/pMB1230 | This study |
| MB1266 | DRH2074/pRY108 | This study |
| MB1268 | DRH3304/pRY108 | This study |
| MB1270 | DRH1407/pRY108 | This study |
| MB1272 | DRH1407/pMB1230 | This study |
| SNJ907 | DRH461 *fliE*::*cat-rpsL* | This study |
| SNJ915 | DRH461 ∆*fliE* | This study |
