# Supplementary material for: Polar Flagellar Biosynthesis and a Regulator of Flagellar Number Influence Spatial Parameters of Cell Division in Campylobacter jejuni
Source: PLoS Pathog. 2011 Dec 1;7(12):e1002420. doi: 10.1371/journal.ppat.1002420 (PMC3228812; doi:10.1371/journal.ppat.1002420)
Supplement: Table S2 — Plasmids used in this study. (DOC) [file ppat.1002420.s008.doc]

**Table S2. Plasmids used in this study**

| **Plasmid** | **Genotype/Description** | **Source/Reference** |
| --- | --- | --- |
| pUC19 | Amp^R^ | New England Biolabs |
| pRY108 | Kan^R^; *E. coli*-*C. jejuni* shuttle vector | [13] |
| pECO101 | Kan^R^; pRY108 with *cat* promoter | [14] |
| pCE107 | Kan^R^; contains *flaA* promoter to drive expression of ZSGreen GFP; contains in-frame BamHI site between promoter and GFP gene to generate protein fusions to the 5’ end of GFP | [15] |
| pDRH265 | pUC19::*cat-rpsL* | [3] |
| pDRH580 | pUC19::*astA-kan* | [1] |
| pDRH532 | pUC19 containing *flgE2*::*nemo* | [1] |
| pDRH642 | pUC19::*fliP* | [1] |
| pDRH655 | pUC19 containing *flaA*::*astA-kan* | [1] |
| pDRH664 | pUC19::*flhA* | [1] |
| pDRH665 | pUC19 containing *flaB*::*astA-kan* | [1] |
| pDRH742 | pUC19::*flhB* | [1] |
| pDRH867 | pDRH664 with *astA-kan* cassette cloned into the NcoI site of *flhA* | This study |
| pDRH1350 | pUC19 with 2.3 kb fragment containing *fliN* from 81-176 cloned into the BamHI site | This study |
| pDRH1367 | pDRH1350 with *cat-rpsL* cassette cloned into the EcoRV site in *fliN* | This study |
| pDRH1454 | pUC19 with 1.7 kb fragment containing *fliQ* from 81-176 cloned into the BamHI site | This study |
| pDRH1814 | pUC19 containing *fliF*::*cat-rpsL* | [4] |
| pDRH2407 | pUC19 with 2.4 kb fragment containing *fliG* from 81-176 cloned into the BamHI site | This study |
| pDRH2428 | pUC19 with 2.6 kb fragment containing the *flgBCfliE* locus of 81-176 cloned into the BamHI site | This study |
| pSMS248 | pUC19 with 2.3 kb fragment containing *flhG* from 81-176 cloned into the BamHI site | This study |
| pSMS259 | pSMS248 with a mutation to generate a MscI site in *flhG* | This study |
| pSMS275 | pSMS259 with *cat-rspL* cassette cloned into the MscI site in *flhG* | This study |
| pSMS279 | pSMS259 with *cat-rpsL* cassette cloned into the MscI site in *flhG* | This study |
| pSMS462 | pDRH1454 with a mutation to generate a MscI site in *fliQ* | This study |
| pSMS469 | pSMS462 with a *cat-rpsL* cassette cloned into the MscI site of *fliQ* | This study |
| pSMS443 | pDRH1454 with an in-frame deletion of codons of 10 -72 of *fliQ* | This study |
| pMB109 | pSNJ128 with an *astA-kan* cassette cloned into the MscI site of *fliP* | This study |
| pMB144 | pDRH742 with an *astA-kan* cassette cloned into the StuI site of *flhB* | This study |
| pMB722 | pCE107 with the coding sequence of *C. jejuni* 81-176 *flhG* from the start to penultimate codons cloned into the BamHI site | This study |
| pMB752 | pSMS248 with an in-frame deletion of codons 14 - 241 of *flhG* | This study |
| pMB865 | pCE107 with the coding sequence of *C. jejuni* 81-176 *flhG* from the start to stop codons cloned into the BamHI site | This study |
| pMB913 | pCE107 with the coding sequence of *H. pylori* J99 *flhG* from the start to stop codons cloned into the BamHI site | This study |
| pMB915 | pCE107 with the coding sequence of *H. pylori* J99 *minD* from the start to stop codons cloned into the BamHI site | This study |
| pMB917 | pCE107 with the coding sequence of *E. coli* MG1655 *minD* from the start to stop codons cloned into the BamHI site | This study |
| pMB957 | pSMS248 with a point mutation to generate *flhG_D61A_* | This study |
| pMB1014 | pCE107 with the coding sequence of *V. cholerae* O395 *flhG* from the start to stop codons cloned into the BamHI site | This study |
| pMB1018 | pCE107 with the coding sequence of *V. cholerae* O358 *minD* from the start to stop codons cloned into the BamHI site | This study |
| pMB1142 | pCE107 with the coding sequence of *C. jejuni* 81-176 *ftsZ* from the start to stop codons cloned into the BamHI site | This study |
| pMB1230 | pECO101 with the coding sequence of *flhG* from codon 2 – stop codon cloned into the BamHI site | This study |
| pJMB532 | pUC19 with 2.5 kb fragment containing *fliM* from 81-176 cloned into the BamHI site | This study |
| pJMB572 | pJMB532 with *cat-rpsL* cassette cloned into the ClaI site of *fliM* | This study |
| pSNJ128 | pDRH642 with a mutation to generate a MscI site in *fliP* | This study |
| pSNJ822 | pDRH2428 with a mutation to generate a StuI site in *fliE* | This study |
| pSNJ918 | pDRH2428 with an in-frame deletion of codons of 2 - 78 of *fliE* | This study |
| pALU115 | pDRH2407 with *cat-rpsL* cassette cloned into the ClaI site of *fliG* | This study |
